# Rapid pathway prototyping and engineering using in vitro and in vivo synthetic genome SCRaMbLE-in methods

**DOI:** 10.1038/s41467-018-04254-0

**Published:** 2018-05-22

**Authors:** Wei Liu, Zhouqing Luo, Yun Wang, Nhan T. Pham, Laura Tuck, Irene Pérez-Pi, Longying Liu, Yue Shen, Chris French, Manfred Auer, Jon Marles-Wright, Junbiao Dai, Yizhi Cai

**Affiliations:** 1 School of Biological Sciences, The King’s Buildings, University of Edinburgh, Edinburgh, EH9 3BF UK; 2 Center for Synthetic Genomics, Institute of Synthetic Biology, Shenzhen Institutes of Advanced Technology, Chinese Academy of Sciences, 518055 Shenzhen, China; 3 BGI-Shenzhen, Beishan Industrial Zone, 518083 Shenzhen, China; 4 China National GeneBank, BGI-Shenzhen, Jinsha Road, 518120 Shenzhen, China; 5 Guangdong Provincial Key Laboratory of Genome Read and Write, Jinsha Road, 518120 Shenzhen, China; 6 Manchester Institute of Biotechnology, University of Manchester, Manchester, M1 7DN UK; 7 Edinburgh Medical School, Biomedical Sciences, The King’s Buildings, Edinburgh, EH9 3BF UK; 8 School of Natural and Environmental Sciences, Devonshire Building, Newcastle University, Newcastle upon, Tyne, NE1 7RX UK

## Abstract

Exogenous pathway optimization and chassis engineering are two crucial methods for heterologous pathway expression. The two methods are normally carried out step-wise and in a trial-and-error manner. Here we report a recombinase-based combinatorial method (termed “SCRaMbLE-in”) to tackle both challenges simultaneously. SCRaMbLE-in includes an in vitro recombinase toolkit to rapidly prototype and diversify gene expression at the pathway level and an in vivo genome reshuffling system to integrate assembled pathways into the synthetic yeast genome while combinatorially causing massive genome rearrangements in the host chassis. A set of loxP mutant pairs was identified to maximize the efficiency of the in vitro diversification. Exemplar pathways of β-carotene and violacein were successfully assembled, diversified, and integrated using this SCRaMbLE-in method. High-throughput sequencing was performed on selected engineered strains to reveal the resulting genotype-to-phenotype relationships. The SCRaMbLE-in method proves to be a rapid, efficient, and universal method to fast track the cycle of engineering biology.

## Abstract (web summary)

Pathway optimization and chassis engineering are usually carried out in a step-wise and trial-and-error manner. Here the authors present ’SCRaMbLE-in’ that combines in-vitro pathway rapid prototyping with in-vivo genome integration and optimization.

## Introduction

One of the exciting recent developments in industrial biotechnology is the synergy between synthetic biology and metabolic engineering to produce fuels, novel medicine and high-value chemicals, nutrition supplements, anti-tumor molecules, and antibiotics^1–3^. In traditional metabolic engineering, metabolic analysis, substrate optimization, and direct genetic modification were often trial-and-error exercises^4^, while synthetic biology provides an opportunity to engineer biology in a more standardized and rational fashion. With the continually dropping cost of DNA synthesis and innovation of DNA assembly methods^5,6^, a wide range of metabolic pathways have been constructed and transformed to heterologous hosts^7–9^. However, introducing a new pathway into a host organism is likely to be accompanied with low production of desired compound molecules due to substrate or co-factor limitations; compromised growth of the host; or even toxicity to the host^10^. A number of methods have been developed to diversify the expression of heterologous pathways, such as the combinatorial assembly of gene expression units, genes expression regulation using CRISPR, protein engineering and pathway compartmentalization^3,11–16^. Despite all the progress that has been made, pathway optimization and chassis engineering remains time- and resource-intensive^3,4^. This is best illustrated by the heroic effort to engineer yeast to produce artemisinin, which required roughly 100 post-doc years^8^.

Site-specific recombinases are a family of DNA modifying enzymes that can recognize and drive recombination between two specific DNA sites to generate deletion, inversion, or integration of DNA fragments between the target sites. The well-studied Cre recombinase recognizes a 34 bp loxP site and drives recombination without any co-factors^17^. The direction of the recombination site determines the type of recombination activity. Site-specific recombinase Cre has been widely applied to gene editing and genome engineering in vivo^18,19^ and the loxP site has also been engineered for better integration efficiency and cassette exchange purposes^17,20–22^. Not only can Cre recombinase drive DNA recombination in vivo, it has also been purified and shown to function in vitro^23^. A number of Cre orthologues, including Dre and VCre, have recently been identified by genome mining and demonstrated to have in vivo function^23–25^. Additionally, Chuang et al.^26^ carried out an extensive mutant library screening, and identified novel rox sites, which can efficiently recombine with themselves in vivo but have minimal to no reactivity with wild-type (WT) rox sites^26^.

Besides the expression level of the target genes, the choice of host and the genetic background of the host is of vital importance for heterologous pathway production^27,28^. Repressing competing pathways and knocking out non-essential genes interacting with the target pathway can re-direct the precursor transformation fluxes in the metabolic maps or remove toxicity of side products to potentially achieve higher target production. However, refactoring the host genome could potentially impact fitness of the cell, and genome-wide chassis optimization is still a daunting endeavor. Traditional mutagenesis and genome-editing technologies such as TALEN- and CRISPR/Cas9-based methods typically introduce a limited number of site-specific mutation(s)^29–31^, but not large-scale genome-wide rearrangements such as inter- and intra-chromosomal translocations. In the synthetic yeast genome Sc2.0 project (www.syntheticyeast.org), the SCRaMbLE system (Synthetic Chromosome Rearrangement and Modification by loxP-mediated Evolution) was designed to introduce genome-wide loxPsym sites and can generate a massive number of genome permutations upon induction^32,33^. SCRaMbLEing the synthetic yeast strains under selective conditions allows efficient directed evolution of desired phenotypes. With continuing successful synthesis and construction of these synthetic chromosomes^18,34–37^, the synthetic yeast is becoming a very attractive platform for metabolic engineering.

In this study, we developed a recombinase-based toolkit “SCRaMbLE-in” to enable rapid metabolic engineering through pathway prototyping and chassis optimization in yeast. SCRaMbLE-in is composed of two steps, which can be used either independently or combined. The first step is to use purified recombinases in vitro to integrate regulatory elements into targeted pathways to diversify gene expression. The second step is to randomly integrate the assembled pathway into the synthetic yeast chromosomes through the SCRaMbLE-in process, which also causes large-scale genome rearrangements. The violacein and β-carotene pathway are used to demonstrate the effectiveness and simplicity of the SCRaMbLE-in method. We also demonstrate that the yield of both pathways can be further optimized with continuous genome SCRaMbLEing.

## Results

### Design of the SCRaMbLE-in system

**Fig. 1:**
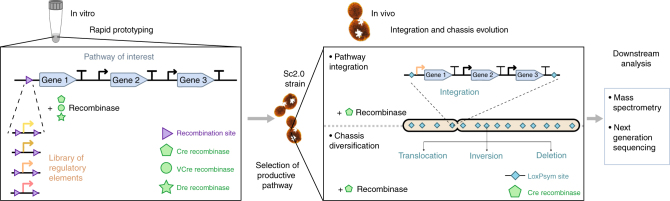
Schematic overview of the SCRaMbLE-in toolkit for metabolic engineering. Once designed, the synthetic pathway of interest will be rapidly prototyped in vitro. The pathway is pre-assembled in such a way that all genes are assembled with respective regulatory elements, except that one key gene is left “promoter-less”, having a recombination site upstream. Three recombination systems, Cre-loxP, VCre-Vlox, and Dre-rox, were developed to integrate a range of promoters upstream to the key gene in the pathway. The assembled library is transformed into yeast and potentially productive pathways are selected and integrated in vivo in the next step. The selected productive pathways are flanked with a pair of loxPsym sites and can subsequently be integrated into the synthetic chromosomes through SCRaMbLE, and simultaneously the synthetic yeast chassis undergoes whole-genome rearrangement to result in strain optimization. The successful integrated strains will be profiled with mass spectrometry analysis and next-generation sequencing

Gene expression diversification and strain optimization are at the core of metabolic engineering. In order to achieve differentiated gene expression and chassis engineering simultaneously, a two-step diversification method was designed (Fig. 1).

Firstly, an in vitro recombinase toolkit was designed to integrate a range of regulatory elements into the pathways of interest (Fig. 1a). In this step, regulatory elements such as promoters are flanked by the recombinase recognition sites in the same orientation. A single recombinase recognition site is engineered into the desired integration position of the pre-assembled pathway. In the case of promoter integration, the target site should be immediately upstream to the key enzyme-coding sequence. Three recombinases, Cre, VCre, and Dre, were purified for use in the integration step. The regulatory elements, selected recombinase and target pathway DNA are mixed in a single reaction, and the recombinase will integrate the regulatory elements into the target recombination site to generate a combinatorially assembled pathway library. The library can then be transformed directly into the yeast for downstream screening and analysis.

Secondly, a SCRaMbLE-in vector has been designed to integrate the target pathway into the synthetic genome through SCRaMbLE (Fig. 1b). In this vector, the target pathway DNA is flanked by the symmetric loxPsym sites. This will allow the Cre recombinase to integrate the target pathway into any loxPsym sites in the synthetic genome. Additionally, the active Cre will also mediate the rearrangement of the synthetic genome, through deletion, inversion, and duplication events, which act to diversify the host genetic background. With proper selection, the host canbe rapidly optimized to better accommodate the heterologous pathway.

### Quantification of the in vitro activity of recombinases

To construct the in vitro recombinase toolkit, we first purified and quantified the activity of three tyrosine recombinases, Cre, Dre, and VCre. The proteins were tagged with C-terminal hexa-histidine tag and purified by nickel affinity chromatography, and subsequently assessed by SDS-PAGE and verified by MALDI peptide mass fingerprinting (Supplementary Fig.1a, b). The test plasmids were designed as shown in Supplementary Fig. 1c. The recombinase will excise the DNA fragment flanked by its recognition sites (Table 1), producing two circular DNA molecules that can be distinguished after ScaI digestion. Therefore, the activity of the recombinase can be monitored by measuring the successful production of the resulting recombined plasmids. We tested the activity of Cre and VCre against both of their recombination sites and found that the two recombinases are orthogonal to each other, i.e., Cre only recombines at the loxP sites and VCre only acts on the VloxP sites (Supplementary Fig. 1e). Furthermore, Dre and VCre are also orthogonal to each other, while Dre has slight cross-activity on loxP sites and is thus not strictly orthogonal to Cre in vitro (Supplementary Fig. 1e).

**Table 1:** Recombinase and target sites

Name	Target site	Length	Site sequence
Cre	loxP	34 bp	ATAACTTCGTATAGCATACATTATACGAAGTTAT
Dre	rox	32 bp	TAACTTTAAATAATTGGCATTATTTAAAGTTA
VCre	Vlox	34 bp	TCAATTTCCGAGAATGACAGTTCTCAGAAATTGA

To quantify the in vitro recombination efficiency, two strategies were designed to measure the excision and integration rates respectively (Supplementary Fig. 1d). The excision rate was assayed by monitoring the loss of an *RFP* gene flanked by a pair of recombinase targeting sites in a reporter plasmid, and defined as the percentage of white colonies on ampicillin selection medium after transforming the reaction mixture into *Escherichia coli*. To measure the integration rate, an accepting vector was constructed, which contains a kanamycin-resistance gene and a single recombination site. In addition, the above-mentioned reporter plasmid was modified to include a *ccdB* gene^38^. Upon recombinase treatment, if the excised *RFP* gene is inserted into the accepting vector, a red colony will be produced on kanamycin-containing medium. The presence of *ccdB* gene prevents the formation of red colonies due to co-transformation of the two original plasmids. The integration rate was defined as the percentage of red colonies after kanamycin selection. As depicted in Supplementary Fig. 1f, the excision rate of Cre-loxP system is over 40%, while VCre-Vlox and Dre-rox systems are slightly lower (about 30%). The relative integration rate is around 0.6% for Cre-loxP and Dre-rox and around 0.3% for VCre-Vlox (Supplementary Fig. 1g). These results indicate that all three recombinases are active in vitro with slightly variable excision and integration rates.

**Fig. 2:**
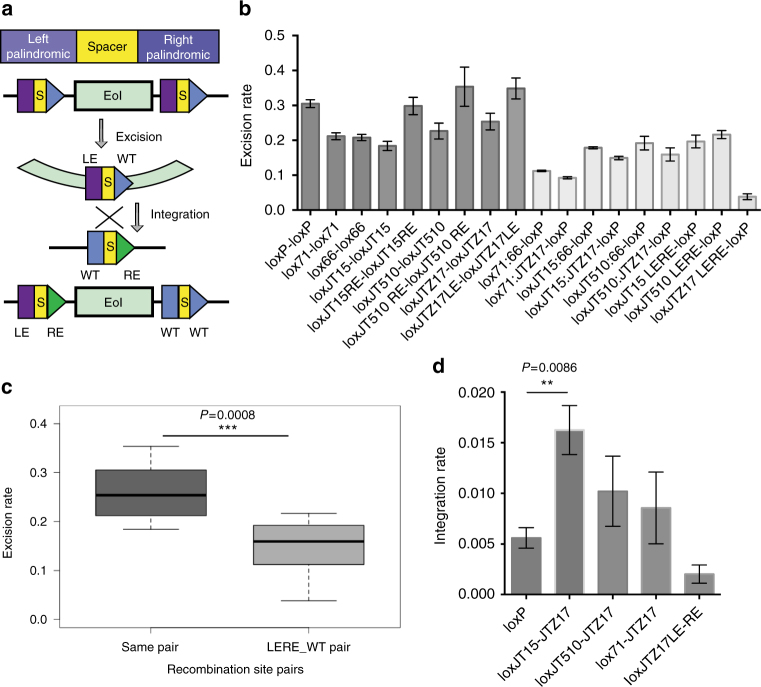
Integration rate improvement by LE/RE strategy. **a** LE/RE strategy was used to improve the integration efficiency. The functional domains of a recombination site are divided as a spacer region and two palindromic regions. The recombination between LE and RE mutation site generates a LE:RE double-mutant site and a wild-type loxP site. For in vitro application, a genetic Element of Interest (EoI) flanked by two LE sites is first excised out, and then integrates into an RE site, finally being flanked by a LE/RE site and a loxP site. **b** Excision rate quantification of palindromic mutant site pairs (LE–LE or RE–RE) and double-mutant site and loxP site (LE:RE-loxP). LE includes lox71, loxJT15, loxJT510, loxJTZ17LE; RE includes lox66, loxJT15RE, loxJT510 RE, loxJTZ17. Error bar represents the standard deviation, *n* = 3. **c** Excision rate comparison between same pair recombination group and LE:RE–WT pair recombination group. Center value represents the mean of the excision rates. *t* test was performed for difference evaluation between the two groups. **d** Integration rate quantification of selected LE- and RE-mutant sites. The integration rate between loxJT15 and loxJTZ17 is three-fold of that between loxP site. Error bar represents the standard deviation and *P* value was generated by *t* test, *n* = 3. LE, left element with mutation in left palindromic region, RE, right element with mutation in right palindromic region

One potential explanation for the low integration rate might be the re-excision of *RFP* cassette from the accepting vector. Therefore, using Cre-loxP as an example, we aimed to increase the rate by introducing mutations into the loxP sites at left or right palindromic arm, termed as left element (LE) and right element (RE)^17^. The loxP site containing both LE and RE could greatly reduce its affinity for the Cre recombinase and consequently preventing the integrated DNA from being excised (Fig. 2a). Five loxP mutants, lox71, lox66, loxJT15, loxJT510, and loxJTZ17, which were reported to increase the integration rate in ES cells and *E. coli* were tested^39,40^. Among them, loxJT15 and loxJT510 only have LE mutation, and loxJTZ17 only has RE mutation. Therefore, we constructed the RE mutation sites of loxJT15 and loxJT510 and the LE mutation site of loxJTZ17 (Table 2). The in vitro excision assay was carried out both between the same loxP mutant sites and between the LE:RE hybrid site and WT site. The result of quantification is summarized in Fig. 2b. In general, we observed that the excision rates of cognate recombination site pairs were higher than that of LE:RE–WT pairs, which suggests the LE/RE strategy is also applicable in vitro (Fig. 2c). Interestingly, loxJTZ17-containing hybrid sites showed the lowest excision rate when recombined with the loxP site, while loxJTZ17 yielded high recombination rate with itself, making it the best candidate for integration improvement. After examining the integration rate, we found that the hybrid site of loxJTZ17 and loxJT15 performs the best, leading to about a three-fold increase relative to the WT site (Fig. 2d). Hence the loxJT15 and loxJTZ17 pair was chosen for next-step application of promoter integration.

**Table 2:** Palindromic arm mutants of loxP

Lox mutant	Mutation element	Sequence
loxP	WT	ATAACTTCGTATAGCATACATTATACGAAGTTAT
lox71	LE	taccgTTCGTATAGCATACATTATACGAAGTTAT
lox66	RE	ATAACTTCGTATAGCATACATTATACGAAcggta
loxJT15	LE	AattaTTCGTATAGCATACATTATACGAAGTTAT
loxJT15 right	RE	ATAACTTCGTATAGCATACATTATACGAAtaatT
loxJT510	LE	taAcgTTCGTATAGCATACATTATACGAAGTTAT
loxJT510 right	RE	ATAACTTCGTATAGCATACATTATACGAAcgTta
loxJTZ17 left	LE	ATAAaTTgcTATAGCATACATTATACGAAGTTAT
loxJTZ17	RE	ATAACTTCGTATAGCATACATTATAgcAAtTTAT

Uppercase indicates wild-type bases; lower case indicates mutation bases. Underline indicates spacer region

*LE* left element, *RE* right element, *WT* wild type

Considering the palindromic structure of recombination sites, their hairpin structure could potentially affect transcriptional or translational efficiency of the downstream target gene after element integration^41^. We examined this effect by using a *GFP* reporter (Supplementary Fig. 2). We integrated three recombination sites, loxP, loxJT15:JTZ17, and Vlox, between three promoters of different strengths and the *GFP* gene. We found that the recombination sites have a similar impact on gene expression regardless of the promoter strength (Supplementary Fig. 2). Of the three recombination sites, the double-mutant site loxJT15:JTZ17 has the minimum impact, which could be due to its asymmetric structure. Therefore, the LE/RE strategy not only improves integration efficiency but also helps to minimize the transcriptional effect of the recombination site on the target gene of interest.

### Pathway prototyping with the in vitro recombinase toolkit

**Fig. 3:**
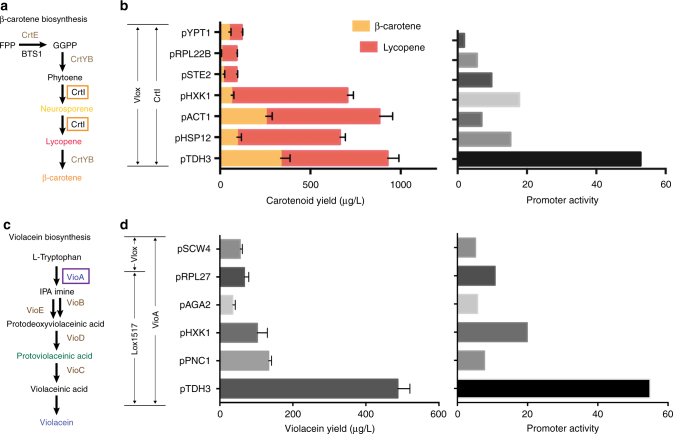
In vitro pathway prototying of β-carotene and violacein pathways. **a** Illustration of gene function in β-carotene synthesis pathway. *CrtI* was chosen as the target gene for integration regulation in β-carotene pathway. *CrtI* encodes the desaturase that converts the colorless phytoene to the yellow neurosporene first and then to the red lycopene through four desaturation reactions. Since it is a key enzyme in catalyzing the colorless intermediates to colored molecules, the extent of yellow, red, or orange can indicate the transformation efficiency and visibly display diversification of the pathway with various expression of *CrtI*. **b** Promoter integration confirmation and LC-MS quantification of carotenoids in β-carotene pathway. VCre-Vlox system was used for *CrtI* regulation. Seven promoter-integrated strains were quantified. Error bar represents the standard deviation, *n* = 3. LC-MS, liquid chromatography and mass spectrometry. **c** Illustration of gene function in violacein synthesis pathway. *VioA* was chosen as the target gene for integration regulation in violacein pathway. *VioA* encodes the flavoenzyme L-tryptophan oxidase that catalyses the incorporation of two molecules of substrate L-tryptophan into indole-3-pyruvic acid imine. It catalyzes the initial step in the violacein synthesis and is important for transforming enough tryptophan substrates for following steps towards full synthesis of the pathway. **d** Promoter integration confirmation and HPLC quantification of violacein. Both VCre-Vlox system and Cre-loxJT15-loxJTZ17 system were used for *VioA* regulation. Six promoter-integrated strains were quantified. Error bar represents the standard deviation, *n* = 3. HPLC, high performance liquid chromatography

The in vitro recombinase toolkit consists of a regulatory element loader plasmid and an acceptor plasmid (Supplementary Fig. 3a). When recombinase is present, the regulatory element will be excised from the loader plasmid and then integrated into the acceptor plasmid to activate and diversify the target gene expression. We have previously reported the construction and characterization of a standardized yeast parts library called YeastFab^10^. To match our in vitro recombinase toolkit with the YeastFab library, several standardizations of the tool plasmids were made. An *RFP* expressing unit flanked by two YeastFab matched type IIs restriction sites was included in the loader to facilitate the loading of standardized elements from YeastFab by Golden Gate assembly and an *URA3* marker was used for selection in yeast (Supplementary Fig. 3b). The acceptor plasmid pWL121 has a similar structure and two homologous arms are included for genome integration at *HO* locus with *HIS3* selection (Supplementary Fig. 3c). We randomly chose 25 promoters in the YeastFab part library with strength varying from 2- to 54-fold relative to that of the pCYC1 promoter as the regulatory elements in this study (Supplementary Fig. 3d, Supplementary Table 1). Based on the integration characterization and *GFP* expression results, Cre-loxJT15:JTZ17 and VCre-Vlox were chosen for pathway diversification. Two exemplar pathways, the β-carotene and violacein synthesis pathways, were selected as demonstration. We selected *CrtI*, the critical gene to transform the colorless phytoene to lycopene in β-carotene synthesis, and *VioA*, the key starting gene transforming L-tryptophan to violacein, as target genes to test promoter integration^42^ (Fig. 3a, c). With the in vitro recombinase reaction, a combinatorial library of β-carotene synthesis pathway with 25 YeastFab promoters driving the *CrtI* gene was generated (Supplementary Fig. 4). The library was then transformed into yeast strain BY4741 and selected on SC-His-Ura plates, which produced yeast colonies of different intensities of orange/yellow colors as well as differing colony sizes, indicating varying yields of carotenoid (Supplementary Fig. 5). The integration of the promoter was confirmed by Sanger sequencing, and the fitness and chemical analysis of several selected variants were further compared (Fig. 3b, d). The yield of carotenoid lycopene and β-carotene were quantified by liquid chromatography-mass spectrometry (LC-MS) and the activity of the integrated promoters was also compared. The result shows that the carotenoid production is not proportional to the activity of the promoters. The medium promoter pACT1 integrated strain have a similar carotenoid yield with pTDH3, while pRPL22B integrated strains have the lowest β-carotene yield even though it is not the weakest promoter (Fig. 3b). Moreover, the result shows different ratios of β-carotene to lycopene are generated (Fig. 3b). Besides difference in yield, the fitness of the integrated strains also varies (Supplementary Fig. 6a). For example, the low yield strain with pYPT1 and pRPL22B have longer doubling time and lower saturation cell densities while the high yield strain with pTDH3, pHSP12, and pACT1 have shorter doubling times and higher saturation point. Although the production levels in strains with pSTE2 or pHXK1 were quite different, the fitness for the two strains is similar. Such fitness differences could be the result of metabolic burden from the intermediates of β-carotene pathway and the flux could be affected by promoter choice.

In contrast, for the violacein pathway, the production of violacein and the activity of the integrated promoters were in proportion with only one exception of pPNC1 (Fig. 3d and Supplementary Fig. 6b). The fitness of the promoter-integrated strains of violacein pathway is similar to each other with the only slower one integrated with pSCW4.

Our results suggest promoter strength does not always correlate with the final pathway production, and the selection will also impact the fitness of the host due to cellular burdens. The in vitro SCRaMbLE-in toolkit allows rapid prototyping of synthetic pathways, and allows the identification of combinations of genetic parts, which can work well together to lead to a productive pathway. The selected pathways can then be stably integrated into the genome in the next step.

### One-step pathway integration and strain evolution

**Fig. 4:**
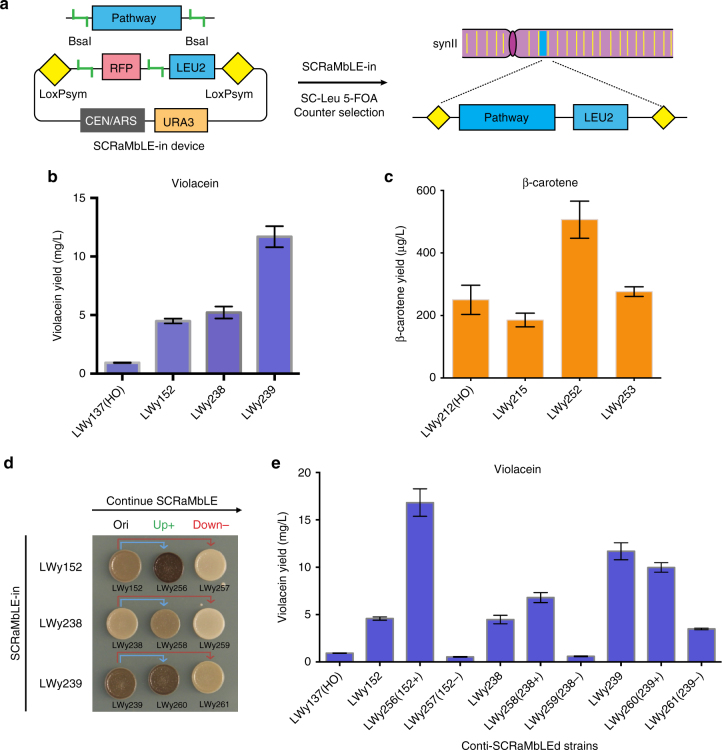
Induction and screening of pathway SCRaMbLE-in yeast variants. **a** A *URA3*-based counter selection strategy to facilitate the SCRaMbLE-in process. The SCRaMbLE-in device is based on a yeast centromeric plasmid with *URA3* marker (pRS416). A BsaI site-flanked *RFP* cassette and a *LEU2* expression cassette were placed between two loxPsym sites. *LEU2* is used as positive selection marker for integration and *URA3* is used as counter selection marker for non-integrated strains. After SCRaMbLE-in induction, successful integrated colonies were selected on SC-Leu + 5-FOA plates. **b** Violacein quantification in the violacein pathway SCRaMbLE-in variants. LWy137 is control strain with a single-copy violacein pathway inserted at the *HO* locus. LWy152, LWy238, and LWy239 are violacein SCRaMbLE-in strains. Error bar represents the standard deviation, *n* = 3. **c** β-carotene quantification in β-carotene pathway SCRaMbLE-in variants. LWy212 is control strain with a single-copy β-carotene pathway inserted at the *HO* locus. LWy215, LWy252, and LWy253 are β-carotene SCRaMbLE-in strains. Error bar represents the standard deviation, *n* = 3. **d** Color comparison of continuous SCRaMbLEd variants with violacein integrated pathway. Both darker colony color and lighter colony color were observed for the three SCRaMbLE-in strains with continuous SCRaMbLE. LWy152, LWy238, and LWy239 are SCRaMbLE-in strains; LWy256 and LWy257 are continuous SCRaMbLEd strains from LWy152; LWy258 and LWy259 are continuous SCRaMbLEd strains from LWy238; LWy260 and LWy261 are continuous SCRaMbLEd strains from LWy239. **e** Violacein quantification of continuous SCRaMbLEd variants with violacein integrated pathway. 152+: increased production compared with source strain LWy152; 152−: decreased production compared with source strain LWy152. Error bar represents the standard deviation, *n* = 3

Besides specific gene regulation and engineering, the genome context can be optimized for the pathway in a synthetic yeast background through our in vivo SCRaMbLE-in method. We chose synthetic chromosome *II*, which contains 410 open-reading frames and 267 loxPsym sites, as the target chromosome for pathway integration^36^. When SCRaMbLE is activated, the pathway can be integrated randomly into the synthetic chromosome with simultaneously random gene deletion, inversion, and duplication of the chromosome. Similar to the in vitro element loader plasmid pWL121, we used a same pair of BsaI sites to flank the *RFP* cassette for the construction of pathway SCRaMbLE-in loading plasmid pWL032 by Golden Gate assembly and a *LEU2* auxotrophic marker for selection (Fig. 4a). The assembly compatibility enables convenient loading of the library pathways generated from in vitro promoter integration for subsequent in vivo genome integration and chassis diversification. When the recombinase is expressed in the synII background, the pathway is first excised out from the *URA3*-marked plasmid and then randomly integrated into the synthetic chromosome. To ensure we only select true integrated strains, 5-FOA counter selection was applied (Fig. 4a and Supplementary Fig. 7a). Genetic compositions of the two pathways are summarized in Supplementary Table 2.

The in vitro result shows that the control strain LWy137 (synII background) with the *HO*-integrated violacein pathway has a yield of around 1 mg/L, while in the SCRaMbLE-in strains LWy152 and LWy238 the yields are around 5 mg/L, almost five-fold more than that of the control strain. The highest yield was identified in the SCRaMbLE-in strain LWy239, which lead to an almost ten-fold increase than that of the control strain (Fig. 4b). For the β-carotene pathway, the total yield of β-carotene for the *HO*-integrated strain LWy212 (BY4741 background) is around 250 μg/L. The total yield of β-carotene of the two SCRaMbLE-in strains LWy215 and LWy253 are similar, but LWy215 is slightly lower than the control, while LWy253 is higher than the control. The total yield of β-carotene for LWy252 is around 500 μg/L, which is twice that of the control strain (Fig. 4c).

Next, we tried to continuously evolve the strains with multiple rounds of SCRaMbLE. Given that the chemical production of the violacein pathway can be roughly estimated from the color of the colony, synII strains with the violacein synthesis pathway integrated were chosen for a continuous SCRaMbLE experiment. Three SCRaMbLE-in strains verified by next-generation sequencing, LWy152, LWy238, and LWy239, were re-transformed with a fresh pSCW11-Cre-EBD plasmid^43^ for continuous SCRaMbLE. After a 24 h induction, the cells were plated onto SC-Leu plates to isolate single colonies. In all three cases of re-SCRaMbLE, we observed clones with darker and lighter colors compared with the original strains, indicating increased and decreased violacein production, respectively (Supplementary Fig. 7b). The colonies were then standardized to OD_600_ = 1.0 at log phase and spotted to SC-Leu to compare the color difference more clearly (Fig. 4d).

After an initial screen by color variation, the composition of violacein and its ratio with the intermediate proviolacein was quantified in the SCRaMbLEd strains. The high-performance liquid chromatography (HPLC) result shows that the peak areas of violacein and proviolacein were diversified among the SCRaMbLEd strains (Supplementary Fig. 8a). LWy256 (LWy152+) has the highest violacein yield of 16.8 mg/L, with more than three-fold that of LWy152 and nearly 17-fold that of the control strain. The yield of LWy258 (LWy238+) is 1.5-fold that of the LWy238 strain. The yield of LWy260 (LWy239+) is similar to that of LWy239. For LWy257 (LWy152−) and LWy259 (LWy238−) with lighter color, their violacein production was reduced to around 0.5 mg/L, which is about 1/10 of the original strains. The yield of violacein in LWy261 (LWy239−) is slightly reduced to 3.5 mg/L. The ratio of violacein to proviolacein in LWy152, LWy257, and LWy259 are similar at around 0.7. In LWy238, LWy258, LWy239, and LWy260, the ratio varies from 1.4 to 1.9. Among these strains, LWy256 has the highest violacein to proviolacein ratio at 3.0 (Supplementary Fig. 8).

### Deep sequencing deconvolution of SCRaMbLE-in strains

By analyzing the PCRtags (Supplementary Fig. 9), we identified that both the LWy257 and LWy259 strains had lost the same pair of PCRTags in the gene *YBR044C*. To investigate whether the lower violacein yields in these two strains was due to *YBR044C* deletion, a gene complementation experiment was performed (Supplementary Fig. 10). The *YBR044C* transcription unit was cloned into a pRS413 vector and transformed into both strains and their parental strains, respectively. The result shows that with the addition of an extra copy of *YBR044C*, both LWy257 and LWy259 recovered to give a color intensity similar to the original LWy152 and LWy238, indicating that the loss of *YBR044C* function contributes to the reduced violacein yield in these strains. The *YBR044C* gene encodes a mitochondrial membrane protein, a putative chaperone involved in the assembly of the succinate dehydrogenase (SDH) complex^44^. The deletion of *YBR044C* did not affect the viability of the cell but could potentially interrupt the function of SDH and further affect the tricarboxylic acid cycle and the mitochondrial respiration chain in *Saccharomyces cerevisiae*. A working hypothesis could be that the violacein synthesis process is NADPH-dependent, and the deletion of *YBR044C* affects the reducing power of the cell, and indirectly affecting the normal NADPH level, which impedes the production of violacein.

**Fig. 5:**
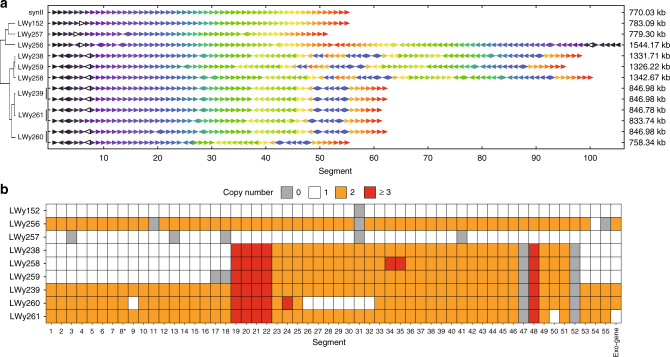
Next-generation sequencing result of violacein SCRaMbLE-in strains. **a** Genome rearrangements in SCRaMbLE-in strains. Each SCRaMbLEd synII chromosome (770 kb) is represented as a sequence of arrows. The color of each arrow represents the segment number separated by loxPsym sites in the parental synII chromosome and the direction indicates the orientation. The unfilled arrow indicates exogenous violacein synthesis pathway. The red-outlined arrow represents the segment where the centromere is located. Based on the SCRaMbLE events, hierarchical clustering method was performed to cluster the strains to illustrate the component differences on the position and length. Remarkably a chromosome fusion event was observed in LWy256 and the total length of SCRaMbLEd synII is 1544.17 kb. **b** Large fragment duplications in the SCRaMbLE-in strains. Segment copy number in each strain is indicated as deletion and no copy (gray), one copy with no change (white), duplication as two copies (orange), and multiple duplications with more than three copies (red). The asterisk represents a CEN2 centromere resides in segment 8

Next-generation sequencing was performed on the SCRaMbLE-in strains. The sequencing results indicate that the entire violacein pathway has been successfully integrated into the synthetic chromosome II (GenBank accession: CP013608.1) at the loxPsym sites at different locations (Fig. 5a and Supplementary Fig. 11). Besides the integrated pathway, many other genome rearrangements in these strains were observed (Fig. 5a and Supplementary Fig. 11), including 26 deletions, 36 inversions, and 32 duplications (Supplementary Data 1). Particularly, large duplications (over 500 kb) were observed in LWy238 and its derived strains LWy259 and LWy258. Whole-chromosome duplication was identified in LWy239 and its derived strains LWy260 and LWy261. One possible explanation that the SCRaMbLE process somehow interferes with the synthetic chromosome segregation process, resulting in one daughter cell with aneuploidy. The largest rearrangement was identified in LWy256 with a length of 1544.17 kb (Fig. 5a and Supplementary Fig. 11), which resulted from a fusion event of two SCRaMbLEd synII chromosomes (Supplementary Fig. 12a). This fusion event leads to the presence of two centromeres in this chromosome, with one of which inverted (Supplementary Fig. 12b). This is supported by the sequencing, PCR and PFGE results (Supplementary Fig.12c, d). The segregation process of this chromosome during cell cycle will be worth studying further, and presumably, one of the centromeres is defective. Considering the complex genotype variations in these strains, it is still difficult to isolate the key variation, or comprehensive effects from numerous variations. Nonetheless, the SCRaMbLE-in and continuous SCRaMbLE processes could provide a platform to generate a rich source of genome rearrangements, which can be further studied with computational and systems biology methods in the future.

### Orthogonal-inducible SCRaMbLE system for future applications

**Fig. 6:**
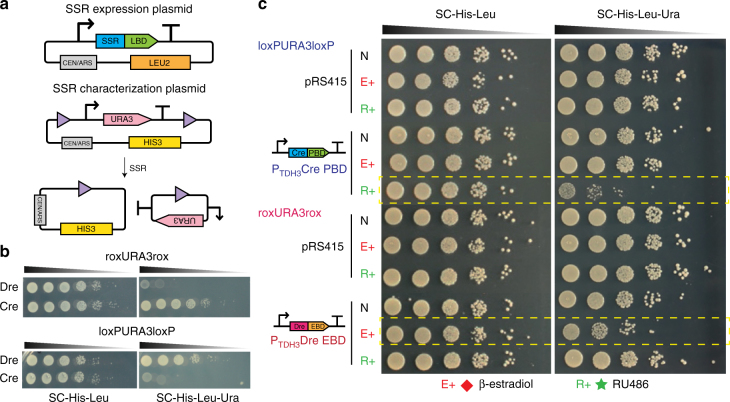
Orthogonal inducible SCRaMbLE systems. **a** Design of SSR expression device and functional test device. SSR or SSR-LBD fusion protein were assembled into a pRS415-based centromeric vector with *LEU2* as selection marker. *URA3* marker was used as recombination reporter and flanked by two recombination sites. The reporter device was based on pRS413 centromeric vector with *HIS3* as selection marker. **b** Orthogonal function test of Cre and Dre. Yeast cells with reporter device rox-*URA3*-rox did not survive with the expression of Dre and cells with reporter device loxP-*URA3*-loxP could not survive with the expression of Cre on SC-His-Leu-Ura plate. **c** Orthogonal induction test of CrePBD and DreEBD. Only cells with reporter device loxP-*URA3*-loxP and CrePBD under the induction of RU486, cells with reporter device rox-*URA3*-rox and Dre-EBD under the induction of β-estradiol did not survive on SC-His-Leu-Ura plate. PBD progesterone-binding domain, EBD estrogen-binding domain, E+ β-estradiol induction, R+ RU486 induction, SSR site-specific recombinase, LBD ligand-binding domain

For further extended applications of recombinases in the synthetic yeast, two controllable orthogonal recombinase expression systems were designed and verified (Fig. 6). The ligand-binding domains (LBDs), including progesterone-binding domain (PBD) and β-estradiol-binding domain (EBD), which have been shown to be able to mediate the translocation of fused protein from the cytoplasm into the nucleus upon ligand binding^43,45^, were used to control the activity of two recombinase orthologues, Cre and Dre. The TDH3 promoter was used to drive the expression of Cre and Dre, with and without the LBDs in a pRS415-based yeast centromeric plasmid, and a reporter plasmid was constructed in a pRS413-based plasmid where a *URA3* expression cassette is flanked by a pair of corresponding recombination sites (Fig. 6a). Both the recombinase expression plasmid and the reporter plasmid were transformed and selected into yeast strain BY4741. The orthogonality of Cre and Dre without LBD fusion was first verified by directly spotting on selective media to assay the recombinase activities (Fig. 6b). Yeast cells with the reporter plasmid rox-*URA3*-rox did not survive with Dre expression and cells with the reporter plasmid loxP-*URA3*-loxP could not survive with Cre expression on SC-His-Leu-Ura plate, proving the activities of the two recombination systems in vivo. The Cre recombinase does not recognize the rox-*URA3*-rox, nor does the Dre recombinase recombines out the loxP-*URA3*-loxP, showing the orthogonality of the two recombinases. We then fused Dre with EBD and Cre with PBD, in order to make the recombinase activities inducible. We demonstrated recombinase activity can be induced by 48 h induction of either 1 μM β-estradiol for DreEBD or 1 μM RU486 for CrePBD. Without the corresponding ligand induction, the recombinase activities are turned off (Fig. 6c). Two rounds of β-estradiol induction were performed as well to achieve a maximal function of DreEBD (Supplementary Fig. 13). In future, the two orthogonal inducible recombination systems can be applied to achieve modular SCRaMbLE/SCRaMbLE-in in either specific regions of the synthetic genome, or even whole-novel-designed synthetic chromosomes, such that more than one pathway can be simultaneously integrated into different genomic loci. The orthogonal modular SCRaMbLE could also facilitate the generation of non-lethal genome rearrangements and evolve the chassis more efficiently for metabolic engineering and other diverse purposes in the Sc2.0 project.

## Discussion

The synergy between synthetic biology and metabolic engineering has attracted more and more attention, along with its successful application in biological materials production. In the past decades, synthetic biology has been widely used in the design, modification and assembly of genetic elements, significantly speeding up the metabolic engineering process, and succeeding in the production of several important metabolites. Recently, great progress has been made in the yeast synthetic genome project—Sc2.0. Six synthetic chromosomes have been successfully synthesized and all are functional in vivo. The synthesized yeast genome offers a unique platform to perform the rapid strain engineering methods such as the SCRaMbLE-in methodologies described in this paper. SCRaMbLEing the synthetic yeast has been proved to be able to generate various genotypes and phenotypes through Cre-mediated rearrangements utilizing the implanted loxPsym sites. In this study, a rapid, efficient, and universal method called SCRaMbLE-in was designed and demonstrated to be a new method to fast track the cycle of engineering biology, presenting a new synergy area between synthetic biology and metabolic engineering.

Combinatorial assembly has been instrumental in synthetic biology for exploring a large design space. Broadly speaking, there are two categories of combinatorial assembly methods: one is based on complementary overhang sequences^5,12,46^, and the other one is using homologous bridging sequences^11,47^. The SCRaMbLE-in utilizes site-specific recombinases to achieve efficient combinatorial library construction, and provides an alternative method to diversify metabolic pathways. It is sequence-independent and can be applied to virtually any parts and pathways. The SCRaMbLE-in method also allows random integration of the pathway into a genomic locus, and at the same time optimize the host Sc2.0 strain, which are not possible with any existing combinatorial methods.

In the SCRaMbLE-in method, the gene expression diversification, pathway integration, and chassis evolution processes were tackled conveniently through our in vitro recombinase toolkit and in vivo synthetic genome SCRaMbLE-in system. The SCRaMbLE-in method not only allows researchers to integrate pathways into the synthetic yeast genome, but also allows the genetic background to be evolved and selected. In addition to the genetic background, the location of integration could affect the transcription efficiency depending on chromatin structure and the degree of heterochromatinization^48–51^. Moreover, the scattered cis-acting elements and other not fully characterized untranslated regions can also make a difference to pathway expression^52^. Though some genomic loci have been studied^51,53^, the majority of the genome remains uncharacterized. The combinatorial nature of the SCRaMbLE-in method allows researchers to explore a large, diversified, genetic space for the optimal design(s). Additionally, the genome-integration feature allows the stable presence of a pathway without generating extra burden of plasmid maintenance in the host. When the heterologous pathway imposes cellular burden to the host, SCRaMbLE provides a unique opportunity for the host to evolve and to adapt to these challenges.

No single strain can serve as a super host for multiple diverse products. Even when a strain is optimized for the production of one class of compound, this rarely translates to enhanced production from different pathways, and so for each target, a lengthy and costly trial-and-error process of strain engineering is required to optimize production. The SCRaMbLE-in method we report here will explore the synthetic yeast, *S. cerevisiae* 2.0 (Sc2.0), as a generic platform for heterologous production of multiple valuable natural products. SCRaMbLE induces genome-wide sequence duplication, deletion, inversion, and translocation, but does not introduce point mutations. Such that it is possible to couple SCRaMbLE with traditional random mutagenesis methods, and recently developed high-throughput genome-wide site-directed mutagenesis methods such as MAGE and RAGE^54,55^.

In our method, pathway SCRaMbLE-in and chassis evolution are achieved in the same step and continuous SCRaMbLE could be applied to further evolve the strains. This makes it particularly attractive for long-term directed evolution experiments using either batch transfer or chemostats. The pathway SCRaMbLE-in vector can serve as a generic tool to integrate virtually any pathway of interest into the synthetic yeast platform. With the merging of multiple synthetic chromosomes and the complete synthesis of the whole yeast genome in near future, the SCRaMbLE-in method will have more use cases to explore.

## Methods

### Protein expression

A single colony of *E. coli* BL21 (DE3), transformed with either the Cre, Dre, or VCre protein expression plasmid, was transferred into 10 mL LB media, supplemented with 100 mg/L kanamycin and incubated overnight at 37 °C with shaking, 180 r.p.m. The overnight pre-culture was then inoculated into 1 L of LB medium with 100 mg/L kanamycin and incubated at 37 °C with 180 r.p.m. shaking. Recombinant protein production was induced when the cell concentration reached 0.6 (OD_600_) by the addition of 1 mM Isopropyl β-D-1-thiogalactopyranoside (IPTG) and the incubation temperature was reduced to 18 °C and cells were incubated for 16 hours. Cells were pelleted by centrifugation at 4000×*g* for 20 min, 4 °C, and resuspended in 10-fold (volume per gram of cell pellet) of PBS to wash cells.

### Protein purification

Cells were resuspended in 10 times (volume per gram of cell pellet) of HisA buffer (50 mM Tris.HCl pH 8.0, 50 mM imidazole, 500 mM NaCl) and lysed by sonication at 4 °C, with ten cycles of 15 s bursts of sonication at 10-μm amplitude followed by 15 s of cooling. The lysate was clarified by centrifugation at 35,000×*g* for 30 min and the supernatant was filtered using a 0.45 μm syringe filter. The filtered supernatant was loaded onto a 5 mL HisTrap FF column equilibrated with HisA buffer. Unbound sample was washed off with 10 column volumes of HisA buffer and protein was eluted with a linear gradient of 0–70% HisB buffer (50 mM Tris.HCl, pH 8.0, 500 mM imidazole, 500 mM NaCl) over 20 column volumes. Peak fractions were analyzed by 15% SDS-PAGE and fractions containing the protein of interest were pooled. Purified Cre, Dre, and VCre recombinase proteins were extensively dialyzed into reaction buffer (50 mM Tris-HCl pH 7.5, 33 mM NaCl, 10 mM MgCl_2_) before use in further assays, or stored by transferring to 50 μL aliquots in thin-walled PCR tubes and flash-cooled in liquid nitrogen before storage at −80 °C. The concentration was adjusted to 10 μM for future use.

### In vitro recombinase quantification assay

For excision rate measurement, 250 ng DNA were mixed with 10 μM recombinase and 5 μL 10X recombination buffer (NEB) in a 50 μL reaction. Keep at 37 °C for 16 h and deactivate recombinase at 75 °C for 15 min. Transform 10 μL into *E. coli* competent cell and select on LB plate with 100 µg/mL ampicillin. For integration rate measurement, 300 ng *RFP*-*AmpR*-*ccdB* plasmid and 100 ng *KanR* plasmid was mixed with 10 μM recombinase and 10 μL 10X recombination buffer in a 100 μL reaction. The reaction was kept at 37 °C for 16 h and the recombinase deactivated at 75 °C for 15 min. *E. coli* competent cells were transformed with 10 µL reaction and selected on an LB + Kanamycin plate. Biological triplicates were performed for rate calculation. Software ImageJ was used to facilitate counting of red and white colonies.

### Promoter library integration by in vitro recombination

Twenty-five promoters from YeastFab library were mixed and assembled with Vlox or loxJTZ15 promoter loading vector by BsmBI Golden Gate assembly. For promoter integration, the recombination reaction was set up by adding 1.5 μg promoter plasmids mixture, 800 ng *HO*-Vlox-*CrtI* β-carotene circuit, or *HO*-loxJTZ15-*VioA* violacein circuit, 10 μL 10X recombination buffer, VCre, or Cre to final concentration of 100 nM, and ddH_2_O to 100 μL. The reaction was kept at 37 °C for 16 h. Frozen competent yeast cells were transformed as follows: The plasmid with promoter-integrated pathway circuit in the reaction was cleaned by PureLink^®^ PCR purification kit and further linearized by BsmBI for homologous recombination at *HO* locus. The reaction was then mixed with 260 μL 50% PEG 3350, 36 μL 1.0 M lithium acetate, 10 μL 10 mg/mL single-stranded carrier DNA and distilled water to a final volume of 360 μL and transformed into 100 μL BY4741 competent cells^56^.

### Hormone induction of CrePBD and DreEBD

Yeast cells were first transformed with a functional test device and selected on SC-His plates, then with the recombinase expressing device pTDH3-CrePBD or pTDH3-DreEBD and selected on SC-His-Leu plates. The fresh double-transformed strain was cultured overnight in non-hormone containing SC-His-Leu liquid medium. The overnight culture was re-inoculated to OD_600_ of 0.1 in SC-His-Leu with 1 µM either β-estradiol (E2257, Sigma-Aldrich) or RU486 (M8046, Sigma-Aldrich) for induction in 30 °C for 48 h. After induction, the cells were adjusted to OD_600_ of 1.0, serial diluted and spotted on SC-His-Leu and SC-His-Leu-Ura plates for comparison.

### In vivo SCRaMbLE-in and variant screening

Yeast with synthetic chromosome II, synII (YSy115), was first transformed with pathway SCRaMbLE-in plasmids, pWL045 and pWL046 for the β-carotene and violacein pathways, respectively, and selected on SC-Leu plates. The strains were further transformed with pRS413-based pSCW11-CreEBD or pTDH3-CreEBD, respectively, and selected on SC-His-Leu plates. Fresh transformants were picked from the plate and cultured in SC-His-Leu liquid media overnight at 30 °C on a roller drum with duplicates for each strain. Cells were inoculated into 10 mL fresh SC-His-Leu liquid medium to OD_600_ of 0.1 with 1 μM estradiol. Cells were cultured for 12, 24, or 48 h at 30 °C. At each time point, serial dilution and spotting were performed on SC-Leu and SC-Leu with 1 mg/mL 5-FOA plates for SCRaMbLE-in efficiency evaluation. For SCRaMbLE-in variant selection, cells were washed and collected after 24 h induction and were spread onto SC-Leu plates with 5-FOA and incubated at 30 °C for 3 days. Variants were selected by color or size and further genotypic verification was performed.

### Violacein extraction and HPLC quantification

The violacein-expressing yeast strain was cultured in corresponding selection media for 5 days at 30 °C. Three mililiters to 5 mL culture was transferred to a 15 mL Falcon tube, spun down at top speed, and washed twice with deionized water. Samples were boiled with 1 mL methanol at 90 °C for 10 min. The debris was spun down at 17,000×*g* for 30 min. The supernatant was transferred to a HPLC glass vial through syringe with needle and 0.22 μm filter. The final volume was standardized to 500 μL or 1 mL by adding more methanol or methanol evaporation.

HPLC analyses were performed on an Agilent 1100 series HPLC system, consisting of a quaternary pump (G1311A), a degasser (G1322A), a FLD detector (G1321A) and a DAD detector (G1315B); column: Agilent ZORBAX SB-C18, 4.6 × 150 mm, 3.5 µm particle diameter size, 80 Å pore size. Analyses were performed using a gradient of A: H_2_O containing 0.1% TFA (trifluoroacetic acid) and B: MeCN (HPLC grade) containing 0.1% TFA with a flow rate of 0.8 mL/min. Gradient used: 0–5 min, 5% B; 5–9 min, 5–25% B; 9–29 min, 25–45% B; 29–40 min, 45–100% B; 40–45 min, 100% B; 45–50 min, 100–5% B; 50–55 min, 5% B. Injection volume for all samples was 25 μL. Retention times (RTs) are denoted in minutes. Absorption values at 210, 220, 256, and 575 nm were recorded. A full absorption spectrum, from 230 to 700 nm, was recorded where peaks were observed in the chromatogram. The quantification methods of violacein are summarized in Supplementary Methods. Chromatograms of commercial violacein and violacein from cell extraction, and violacein calibration curve were generated after HPLC analysis (Supplementary Fig. 14a–d).

### Carotenoid extraction and LC-MS quantification

The yeast strains were cultured in 200 mL flasks at 30 °C 220 r.p.m. for 48 h. The OD_600_ of all the yeast strains was recorded and 10 mL of cell culture was collected by centrifugation at 15,000×*g* for 1 min and washed twice with deionized water. The cells were broken with glass beads (1 g, 0.50–0.75 mm) by vortexing for 3 min, then 600 μL sorbitol (1 M) and 25 μL lyticase (25 mg/mL) were added. The mixture was vortexed for 10 min, followed by 1 h incubation at 37 °C at 220 r.p.m. Pyrogallol of 1.25 mL 0.2% (wt/vol) dissolved in methanol was added and mixed by vortexing for 10 s. KOH of 700 μL 60% (wt/vol) was added for saponification and vortexed for 10 s, followed by 1 h incubation at 75 °C with vortexing every 15 min. The carotenoid fraction was extracted with 5–10 mL hexane, by mixing and left at room temperature overnight with the instrument vertical mixing apparatus. The extraction mixture was centrifuged at 3000×*g* for 5 min. The hexane layer was transferred to new Eppendorf tubes and vacuum-dried for 30 min at 60 °C. The cell pellets were repeated with hexane extraction. The vacuum-dried extractions were re-dissolved in isopropanol and the whole metabolite was cleared with ultrasonication. The cell debris was spun down and the supernatant was transferred to a new tube, followed by lyophilization. The sample pellet was finally resuspended with 100 μL isopropanol and 2 μL of sample was injected into LC-MS equipment for chemical analysis. Separation of lycopene and β-carotene and chemical analysis were performed by the ACQUITY UPLC I-Class system (Waters) and the QTRAP 4500LC-MS/MS system (SCIEX). The column for lycopene and β-carotene separation is ACQUITY UPLC BEH C18 Column, 130 Å, 1.7 µm, 2.1 × 50 mm. Analyses were performed using a gradient of A: 100% methanol and B: 50% methanol and 50% isopropanol with a flow rate of 0.3 mL/min. Gradient used: 0–0.5 min, 30% B; 0.5–1 min, 98% B; 1–4 min, 98% B; 4–4.1 min, 30% B; 4.1–5 min, 30% B. RTs are denoted in minutes. The parameters for MS are as follows. λ source/gas parameters: ion spay voltage: 5500; curtain gas: 35, nebulizer gas (gas 1) 55, ion source GS2 (gas 2) 55; interface heater temperature: 550. λ compound parameters: de-clustering potential: 30; entrance potential: 10; collision cell exit potential: 10. The quantification methods of carotenoids are summarized in Supplementary Methods. Chromatograms of commercial lycopene and β-carotene, and corresponding calibration curves were generated after LC-MS analysis (Supplementary Fig. 14e–g).

### Next-generation sequencing and variation reconstruction

The genomic DNA library was prepared for whole-genome sequencing according to the BGI’s standard preparation protocol using the MGIEasy™ DNA Library Prep Kit V1. One microgram of sample DNA was sheared using a Covaris LE220 to an average length of 350 bp. After end-repairing and adapter ligation with barcode, it was PCR-amplified and -purified. Subsequently, the PCR products with different barcodes were pooled together at equimolar concentration. Sixty microliters of the pool was heat-denatured, and mixed with an equal volume of circularization reaction buffer, which contains 10 μL splint oligo, 12 μL splint buffer, 0.4 μL ligation enzyme, 1.2 μL ligation enhancer, and 36.4 μL nuclease-free water. The mixture was subsequently incubated at 37 °C for 60 min. Finally, 20 μL of each single-circle-library pool was further used to prepare the DNA Nanoballs (DNB) and sequenced on the BGISEQ-500 platform using BGISEQ-500 high-throughput sequencing kit (PE100).

Before mapping to genome, quality control of sequencing reads was performed. Reads with length shorter than 100 bp or >1% of bases that have a Phred-based quality lower than 10 or with unknown bases were removed. These cleaned reads were mapped to synthetic yeast genome sequences using “Bowtie2” (version 2.0.0) with standard parameters^57^. A similar approach to a previously published method that combines both sequencing depth and split-read mapping was performed to identify complex structural variation and SCRaMbLE events^33^. Reads that were mapped to the reference were used to evaluate the sequencing depth by “SAOPcoverage” (version 2.7.7). Reads that did not map to the reference were split into pairwise ends with length of longer than 30 bp by scanning over all possible intermediate positions. These pairwise ends were further aligned to the reference using “Bowtie2”^57^. Combining the sequencing depth and split-read mapping, the structural variations were reconstructed as described.

### Data availability

All data and genetic material used for this paper are available from the authors on request. The sequencing data of the SCRaMbLEd strains have been deposited in the European Nucleotide Archive (ENA) under accession PRJEB23542.

## Electronic supplementary material

Supplementary Information

Description of Additional Supplementary Files

Supplementary Data 1

## References

[1] Yadav VG, De Mey M, Lim CG, Ajikumar PK, Stephanopoulos G (2012). The future of metabolic engineering and synthetic biology: towards a systematic practice. Metab. Eng..

[2] Stephanopoulos G (2012). Synthetic biology and metabolic engineering. ACS Synth. Biol..

[3] Keasling JD (2012). Synthetic biology and the development of tools for metabolic engineering. Metab. Eng..

[4] Nielsen J, Keasling JD (2011). Synergies between synthetic biology and metabolic engineering. Nat. Biotechnol..

[5] Martella A, Matjusaitis M, Auxillos J, Pollard SM, Cai Y (2017). EMMA: an extensible mammalian modular assembly toolkit for the rapid design and production of diverse expression vectors. ACS Synth. Biol..

[6] Colloms, S. D. et al. Rapid metabolic pathway assembly and modification using serine integrase site-specific recombination. *Nucleic Acids Res.* **42**, e23 (2014). 10.1093/nar/gkt1101 PMC3936721 24225316

[7] Thodey K, Galanie S, Smolke CD (2014). A microbial biomanufacturing platform for natural and semisynthetic opioids. Nat. Chem. Biol..

[8] Paddon CJ, Keasling JD (2014). Semi-synthetic artemisinin: a model for the use of synthetic biology in pharmaceutical development. Nat. Rev. Microbiol..

[9] DeLoache WC (2015). An enzyme-coupled biosensor enables (S)-reticuline production in yeast from glucose. Nat. Chem. Biol..

[10] Pitera DJ, Paddon CJ, Newman JD, Keasling JD (2007). Balancing a heterologous mevalonate pathway for improved isoprenoid production in *Escherichia coli*. Metab. Eng..

[11] Mitchell LA (2015). Versatile genetic assembly system (VEGAS) to assemble pathways for expression in *S. cerevisiae*. Nucleic Acids Res..

[12] Guo Y (2015). YeastFab: the design and construction of standard biological parts for metabolic engineering in *Saccharomyces cerevisiae*. Nucleic Acids Res..

[13] Cuperus JT, Lo RS, Shumaker L, Proctor J, Fields S (2015). A tetO toolkit to alter expression of genes in *Saccharomyces cerevisiae*. ACS Synth. Biol..

[14] Zalatan JG (2015). Engineering complex synthetic transcriptional programs with CRISPR RNA scaffolds. Cell.

[15] Dueber JE (2009). Synthetic protein scaffolds provide modular control over metabolic flux. Nat. Biotechnol..

[16] Avalos JL, Fink GR, Stephanopoulos G (2013). Compartmentalization of metabolic pathways in yeast mitochondria improves the production of branched-chain alcohols. Nat. Biotechnol..

[17] Araki K. & Yamamura K. I. in. *Controlled Genetic Manipulations. Neuromethods*, Vol. **65** (ed. Morozov, A.) 29–45 (Humana Press, Totowa, NJ, 2012).

[18] Annaluru N (2014). Total synthesis of a functional designer eukaryotic chromosome. Science.

[19] Livet J (2007). Transgenic strategies for combinatorial expression of fluorescent proteins in the nervous system. Nature.

[20] Missirlis PI, Smailus DE, Holt RA (2006). A high-throughput screen identifying sequence and promiscuity characteristics of the loxP spacer region in Cre-mediated recombination. BMC Genomics.

[21] Sheren J, Langer SJ, Leinwand LA (2007). A randomized library approach to identifying functional lox site domains for the Cre recombinase. Nucleic Acids Res..

[22] Bouhassira EE, Westerman K, Leboulch P (1997). Transcriptional behavior of LCR enhancer elements integrated at the same chromosomal locus by recombinase-mediated cassette exchange. Blood.

[23] Ghosh K, Van Duyne GD (2002). Cre-loxP biochemistry. Methods.

[24] Sauer B, McDermott J (2004). DNA recombination with a heterospecific Cre homolog identified from comparison of the pac-c1 regions of P1-related phages. Nucleic Acids Res..

[25] Minorikawa S, Nakayama M (2011). Recombinase-mediated cassette exchange (RMCE) and BAC engineering via VCre/VloxP and SCre/SloxP systems. Biotechniques.

[26] Chuang K, Nguyen E, Sergeev Y, Badea TC (2015). Novel heterotypic Rox sites for combinatorial Dre recombination strategies. G3.

[27] Jouhten P (2016). Yeast metabolic chassis designs for diverse biotechnological products. Sci. Rep..

[28] Lee SK, Chou H, Ham TS, Lee TS, Keasling JD (2008). Metabolic engineering of microorganisms for biofuels production: from bugs to synthetic biology to fuels. Curr. Opin. Biotechnol..

[29] Jung WS (2011). A combined approach of classical mutagenesis and rational metabolic engineering improves rapamycin biosynthesis and provides insights into methylmalonyl-CoA precursor supply pathway in *Streptomyces hygroscopicus* ATCC 29253. Appl. Microbiol. Biotechnol..

[30] Jakociunas T, Jensen MK, Keasling JD (2016). CRISPR/Cas9 advances engineering of microbial cell factories. Metab. Eng..

[31] Wood AJ (2011). Targeted genome editing across species using ZFNs and TALENs. Science.

[32] Dymond JS (2011). Synthetic chromosome arms function in yeast and generate phenotypic diversity by design. Nature.

[33] Shen Y (2016). SCRaMbLE generates designed combinatorial stochastic diversity in synthetic chromosomes. Genome Res..

[34] Mercy, G. et al. 3D organization of synthetic and scrambled chromosomes. *Science* **355**, eaaf4597 (2017). 10.1126/science.aaf4597 PMC5679085 28280150

[35] Richardson SM (2017). Design of a synthetic yeast genome. Science.

[36] Shen, Y. et al. Deep functional analysis of synII, a 770-kilobase synthetic yeast chromosome. *Science* **355**, eaaf4791 (2017). 10.1126/science.aaf4791 PMC5390853 28280153

[37] Dai, J., Cai, Y., Yuan, Y., Yang, H. & Boeke, J. D. Whole genome synthesis: from poliovirus to synthetic yeast. *Quant. Biol.* **5**, 105–109 (2017).

[38] Bernard P (1993). The F plasmid CcdB protein induces efficient ATP-dependent DNA cleavage by gyrase. J. Mol. Biol..

[39] Araki K, Araki M, Yamamura KI (1997). Targeted integration of DNA using mutant lox sites in embryonic stem cells. Nucleic Acids Res..

[40] Thomson JG, Rucker EB, Piedrahita JA (2003). Mutational analysis of loxP sites for efficient Cre-mediated insertion into genomic DNA. Genesis.

[41] Kudla G, Murray AW, Tollervey D, Plotkin JB (2009). Coding-sequence determinants of gene expression in *Escherichia coli*. Science.

[42] Verwaal R (2007). High-level production of beta-carotene in *Saccharomyces cerevisiae* by successive transformation with carotenogenic genes from *Xanthophyllomyces dendrorhous*. Appl. Environ. Microbiol..

[43] Lindstrom DL, Gottschling DE (2009). The mother enrichment program: a genetic system for facile replicative life span analysis in *Saccharomyces cerevisiae*. Genetics.

[44] Dibrov E, Fu S, Lemire BD (1998). The *Saccharomyces cerevisiae* TCM62 gene encodes a chaperone necessary for the assembly of the mitochondrial succinate dehydrogenase (complex II). J. Biol. Chem..

[45] Anastassiadis K (2009). Dre recombinase, like Cre, is a highly efficient site-specific recombinase in *E. coli*, mammalian cells and mice. Dis. Model Mech..

[46] Agmon N (2015). Yeast golden gate (yGG) for the efficient assembly of *S. cerevisiae* transcription units. ACS Synth. Biol..

[47] Trubitsyna M, Michlewski G, Cai Y, Elfick A, French CE (2014). PaperClip: rapid multi-part DNA assembly from existing libraries. Nucleic Acids Res..

[48] Mano Y, Kobayashi TJ, Nakayama J, Uchida H, Oki M (2013). Single cell visualization of yeast gene expression shows correlation of epigenetic switching between multiple heterochromatic regions through multiple generations. PLoS Biol..

[49] Kitada T (2012). Mechanism for epigenetic variegation of gene expression at yeast telomeric heterochromatin. Gene Dev..

[50] Ay F (2014). Three-dimensional modeling of the *P. falciparum* genome during the erythrocytic cycle reveals a strong connection between genome architecture and gene expression. Genome Res..

[51] Englaender, J. A. et al. Effect of genomic integration location on heterologous protein expression and metabolic engineering in *E. coli*. *ACS Synth. Biol.* **6**, 710–720 (2017). 10.1021/acssynbio.6b00350 28055177

[52] Yoshikawa K, Furusawa C, Hirasawa T, Shimizu H (2008). Genome-wide analysis of the effects of location and number of stress response elements on gene expression in *Saccharomyces cerevisiae*. J. Biosci. Bioeng..

[53] Flagfeldt DB, Siewers V, Huang L, Nielsen J (2009). Characterization of chromosomal integration sites for heterologous gene expression in *Saccharomyces cerevisiae*. Yeast.

[54] Wang HH (2009). Programming cells by multiplex genome engineering and accelerated evolution. Nature.

[55] Santos CN, Regitsky DD, Yoshikuni Y (2013). Implementation of stable and complex biological systems through recombinase-assisted genome engineering. Nat. Commun..

[56] Gietz RD, Schiestl RH (2007). Frozen competent yeast cells that can be transformed with high efficiency using the LiAc/SS carrier DNA/PEG method. Nat. Protoc..

[57] Langmead B, Trapnell C, Pop M, Salzberg SL (2009). Ultrafast and memory-efficient alignment of short DNA sequences to the human genome. Genome Biol..

