# Supplementary material for: Rapid pathway prototyping and engineering using in vitro and in vivo synthetic genome SCRaMbLE-in methods
Source: Nat Commun. 2018 May 22;9:1936. doi: 10.1038/s41467-018-04254-0 (PMC5964202; doi:10.1038/s41467-018-04254-0)
Supplement: Supplementary file 1 — Supplementary Information [file 41467_2018_4254_MOESM1_ESM.pdf]

Supplementary Information

Rapid pathway prototyping and engineering using *in vitro* and *in vivo* synthetic genome SCRaMbLE-in methods

Wei *et al.*

List of Content

Supplementary Figures 1-14

Supplementary Tables 1-2

Supplementary Methods

Supplementary References

Supplementary Figures

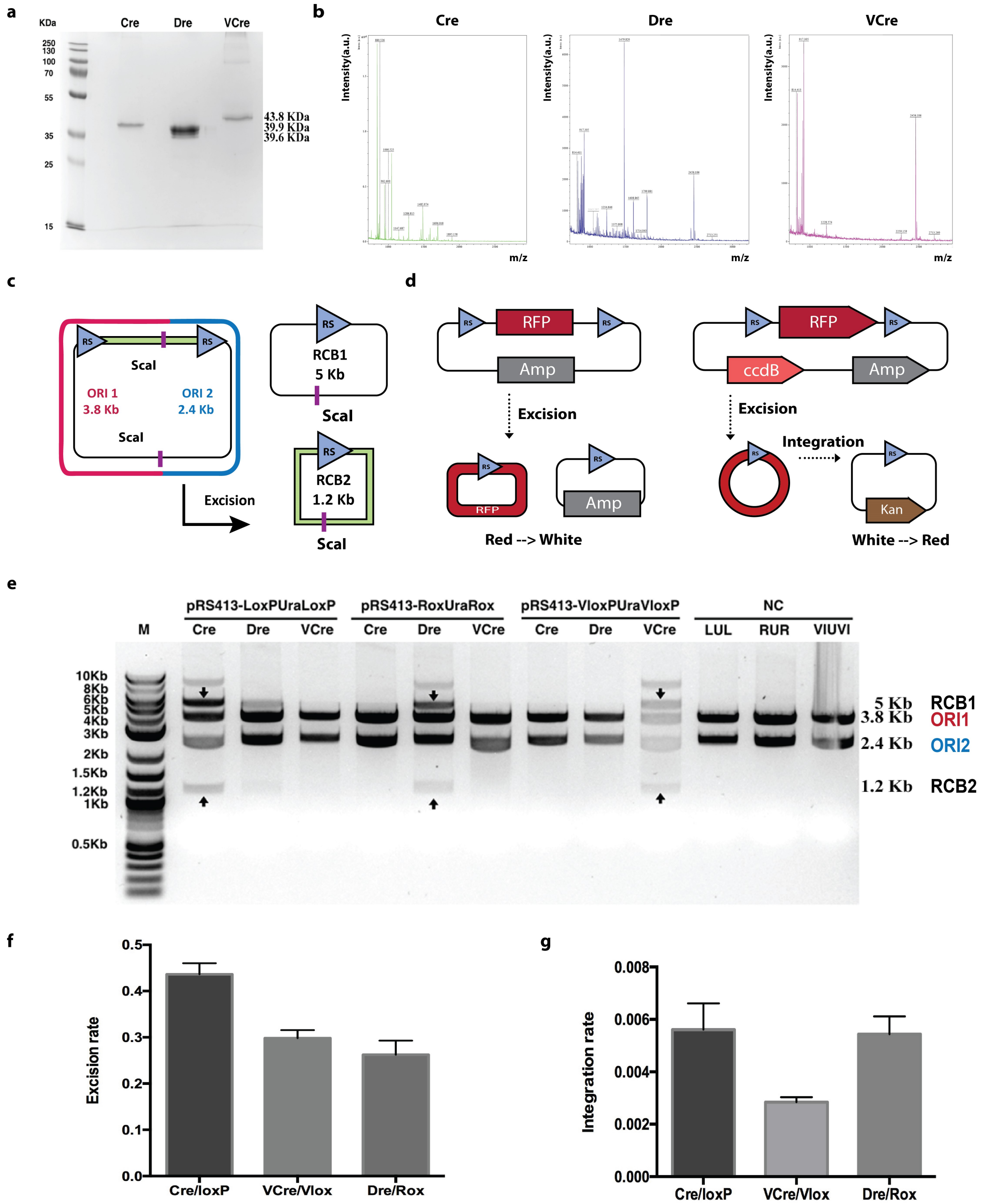

Supplementary Figure 1. Recombinase purification and in vitro function characterization. (a) SDS-PAGE gel of the purified recombinases. The molecular weight of Cre, Dre and VCre are 39.6 KDa, 39.9 KDa and 43.8 KDa. **(b)** MALDI peptide mass fingerprinting of Cre, Dre and VCre. **(c)** *In vitro* function test strategy by DNA digestion pattern variation. ScaI linearization of original plasmid generated two bands (ORI1, 3.8Kb; ORI2, 2.4Kb); ScaI linearization of two circular recombined DNA generates fragments with different sizes (RCB1, 5Kb; RCB 2, 1.2Kb). RCB: recombined band; ORI: original band. **(d)** Excision and integration quantification strategy. The *RFP* gene was used as a recombination indicator. Excision result in changing of the colony color from red to white; integration results in changing the colony color from white to red. Respective antibiotic markers and a *ccdB* counter

selection marker were used to get rid of background plasmids. **(e)** *In vitro* function test result by DNA digestion pattern variation. Recombined bands (RCB1 and RCB2) were indicated by black arrows. The three recombination system are basically orthogonal *in vitro* except that Dre has slight leakiness on *loxP* site. **(f)** Excision rate comparison of Cre, Dre and VCre. Error bar represents the standard deviation (n=3). **(g)** Integration rate comparison of Cre, Dre and VCre. Error bar represents the standard deviation (n=3).

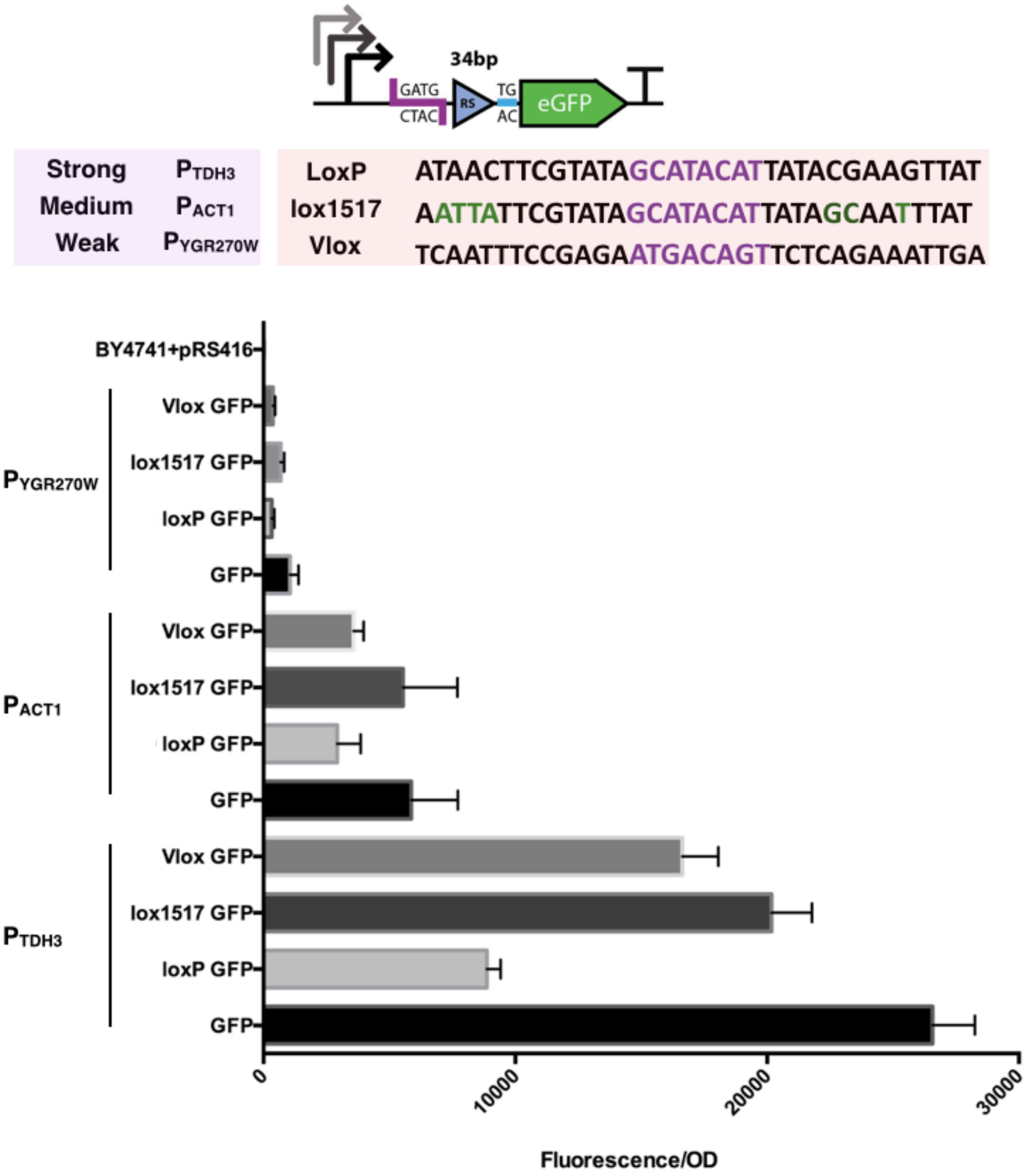

Supplementary Figure 2. Effect of recombination site on gene expression. Three different promoters were used to compare site effect on the expression of GFP. *LoxP*, *lox1517* and *Vlox* were put between the promoter and start codon of GFP. The result shows that the effect of site varies between different site with *lox1517<Vlox<loxP*. The patterns of each site under the three promoters are similar. The fluorescence and cell density OD_{600} were measured by plate reader. Error bar represents standard deviation (n=3). Purple indicates spacer region of the recombination site; green indicates base mutation in the *lox1517* double arm mutant.

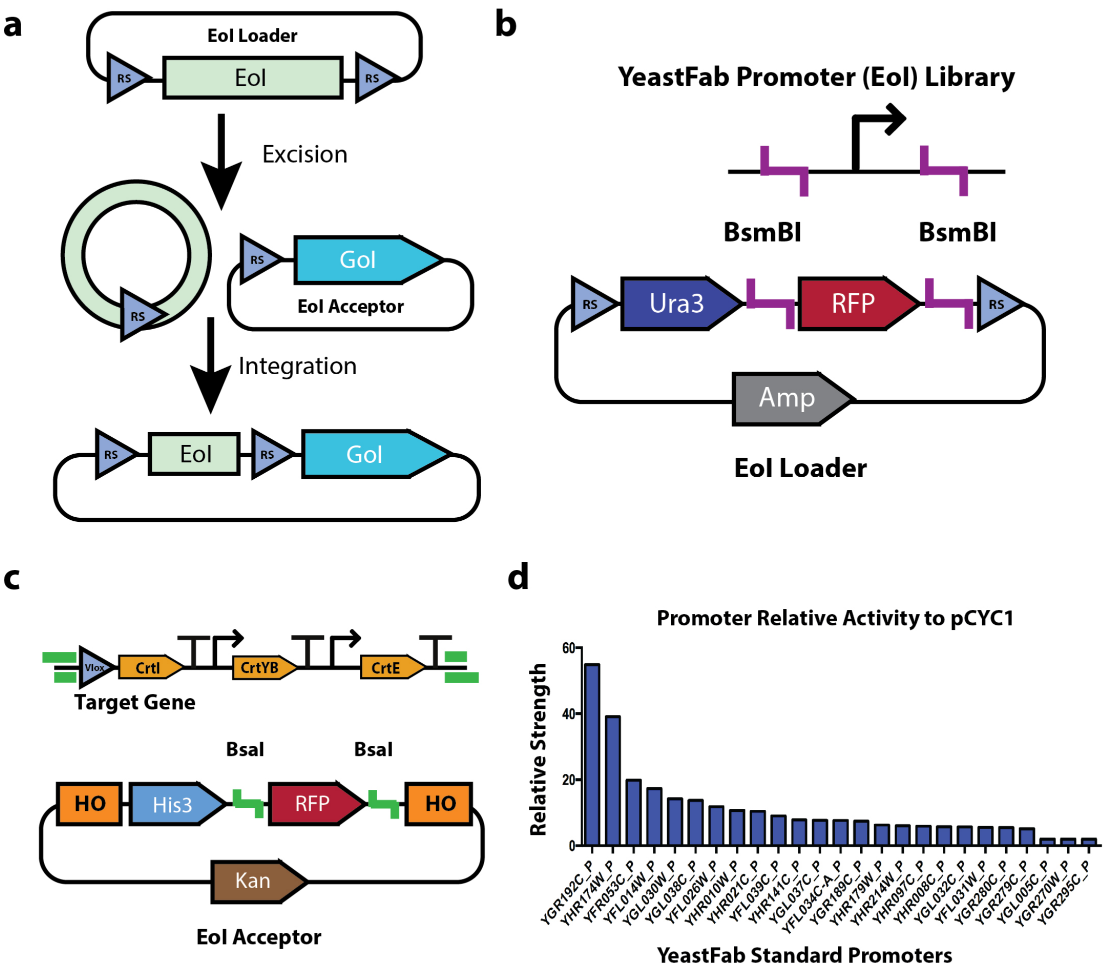

Supplementary Figure 3. Workflow and design of key vectors for *in vitro* integration (a) Workflow of the *in vitro* recombinase toolkit. The element of interest (EoI), which is flanked by two recombination sites, will first go through one step excision with a single site left on EoI. Another recombination site is placed upstream of the gene of interest (GoI). The site on excised EoI will secondly interact with the site upstream GoI to accomplish integration process. **(b)** Design of EoI loading device. The EoI loader is composed of an *URA3* cassette and *RFP* cassette flanked by recombination sites with the ampicillin resistant gene marker. *RFP* cassette is flanked by YeastFab compatible BsmBI site to facilitate the loading of YeastFab promoter library. **(c)** Design of EoI acceptor. The EoI acceptor consists of a *HIS3* cassette and *RFP* cassette. The *RFP* cassette is flanked by YeastFab compatible BsaI site to accommodate genes from targeted pathway. Outside the two cassettes are two homologous arms of *HO* locus for pathway integration to yeast genome. Kanamycin resistant gene is for *in vitro* integration selection and *HIS3* marker is for genome integration selection. **(d)** The strength of selected promoters from YeastFab promoter library, with activities varying from 2 to 54 folds relative to pCYC1.

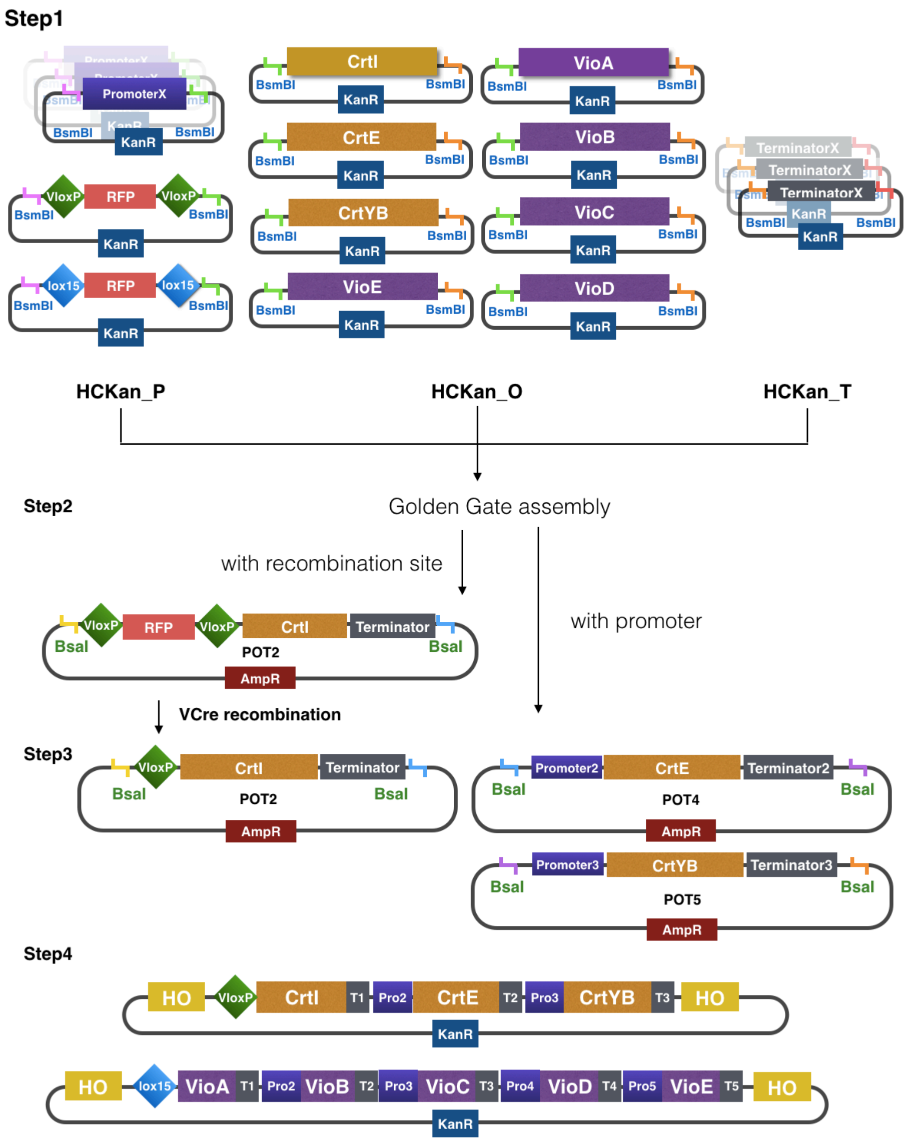

Supplementary Figure 4. Construction strategy of recombination site containing pathway circuit. The construction of pathway circuit includes three rounds of Golden Gate assembly. Step1: recombination sites flanking *RFP* and constitutive promoters were put into HCKan_P receptor vector; *CrtI*, *CrtE*, *CrtYB*, *VioA-E* coding sequences were put into HCKan_O receptor vector; terminators were put into HCKan_T vector. Step2: *RFP*/promoter element, CDS and terminator were assembled into POT unit in different vectors by BsmBI Golden Gate assembly. Step3: the unit with recombination site flanking RFP went through one round of *in vitro* excision by recombinase; unit with constitutive promoter will skip to step4. Step4: POT expression units and recombination site containing units were inserted into final receptor vector by BsaI Golden Gate assembly. All BsmBI sites and BsaI sites were YeastFab standard. The final assembly for violacein pathway with recombination site was shown in step 4 with step 2 and step 3 omitted.

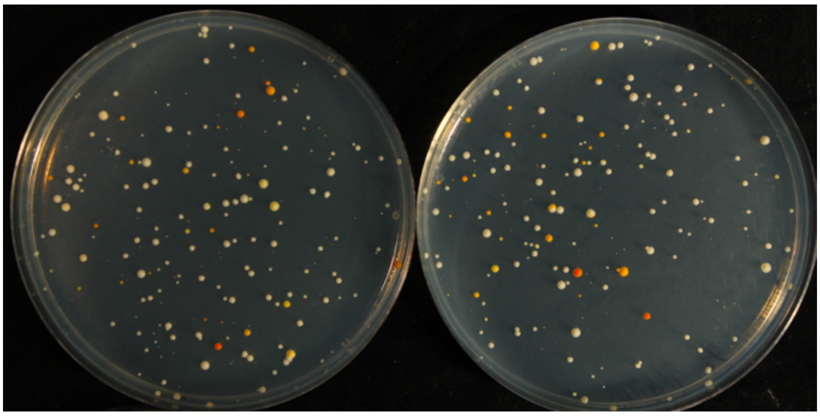

Supplementary Figure 5. Diversity of β-carotene pathway with promoter *in vitro* integration. After promoter integration to *Vlox-CrtI* by VCre recombinase *in vitro*, the reaction was transformed to BY4741 and the transformants were screened on SC-His-Ura plate. After 3 days culture at 30°C, various colony sizes and colors from yellow to orange were observed.

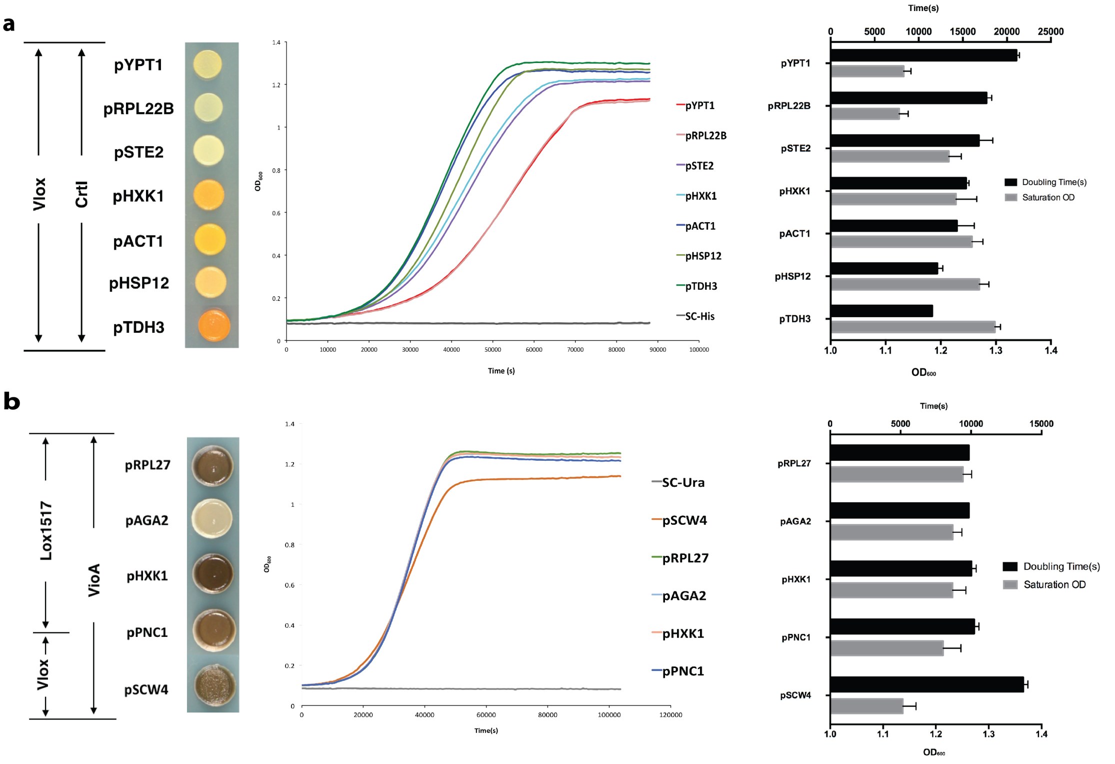

Supplementary Figure 6. Fitness comparison between promoter integrated strains **(a)** Fitness comparison between strains with the promoter integrated β-carotene pathway. The low production strains with pYPT1 and pRPL22B have longer doubling time and lower saturation cell density; the high production strains with pTDH3, pHSP12 and pACT1 have shorter doubling time and higher saturation point. The production of strains with pSTE2 and with pHXK1 were quite different, the fitness for the two strains are similar. **(b)** Fitness comparison between strains with the promoter integrated with violacein pathway. The fitness of the promoter integrated strains of violacein pathway is similar to each other with only one slower strain integrated with pSCW4.

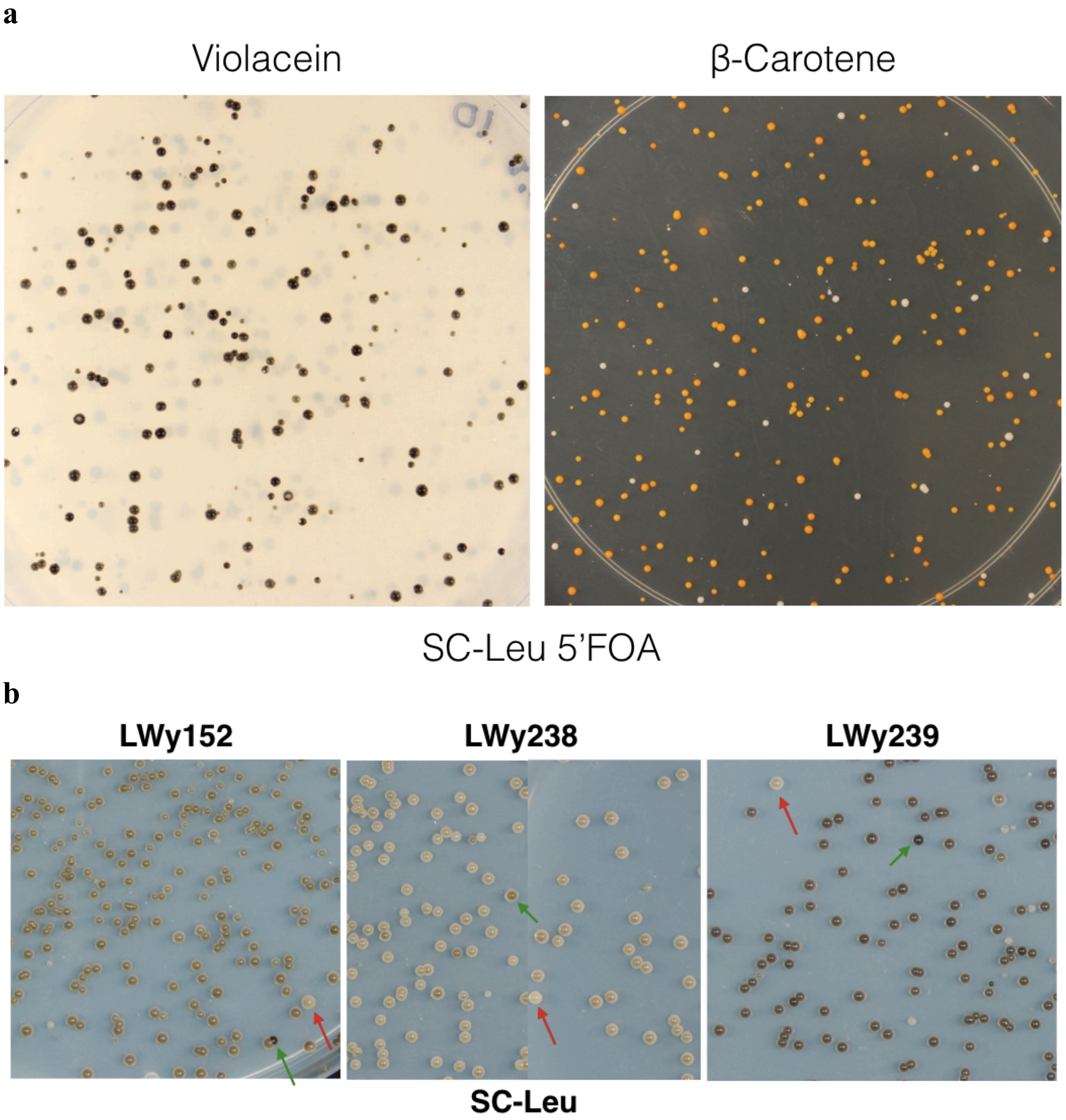

Supplementary Figure 7. Pathway diversity generated by SCRaMbLE-in and continuous SCRaMbLE. (a) SC-Leu 5'FOA selection of violacein and β-carotene SCRaMbLE-in candidates. Diversified colony colors and sizes were observed for both pathways. **(b)** Cell plating screening of SCRaMbLEd strains. Darker colonies were pointed with green arrow and lighter colonies were pointed with red arrow.

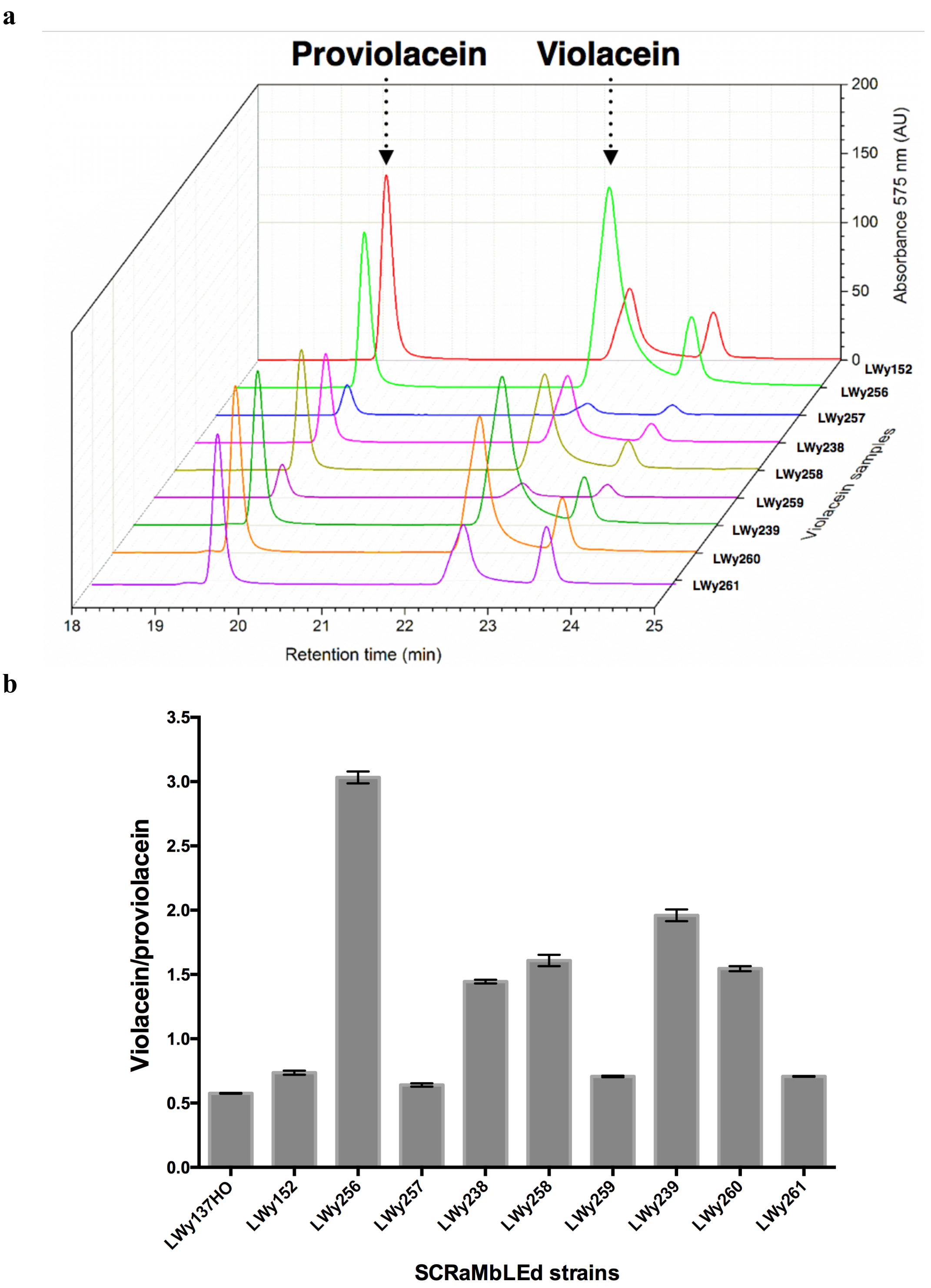

Supplementary Figure 8. HPLC analysis of violacein production of continuous SCRaMbLEd strains. (a) HPLC chromatograms of multiple continuous SCRaMbLEd strains with HPLC. The graph was plotted for multiple strains for easier comparison of the chemical compositions between the mutants. The absorbance was measured at 575 nm, which is the maximum absorption wavelength for violacein. **(b)** Quantification of violacein/proviolacein ratio. The conversion efficiency of proviolacein to violacein was diversified by continuous SCRaMbLE. Strain LWy256 was upregulated from the LWy152 strain after continuous SCRaMbLE and has the highest violacein/proviolacein ratio in addition to the highest violacein yield. Biological replicates (n=3) were performed for the HPLC quantification.

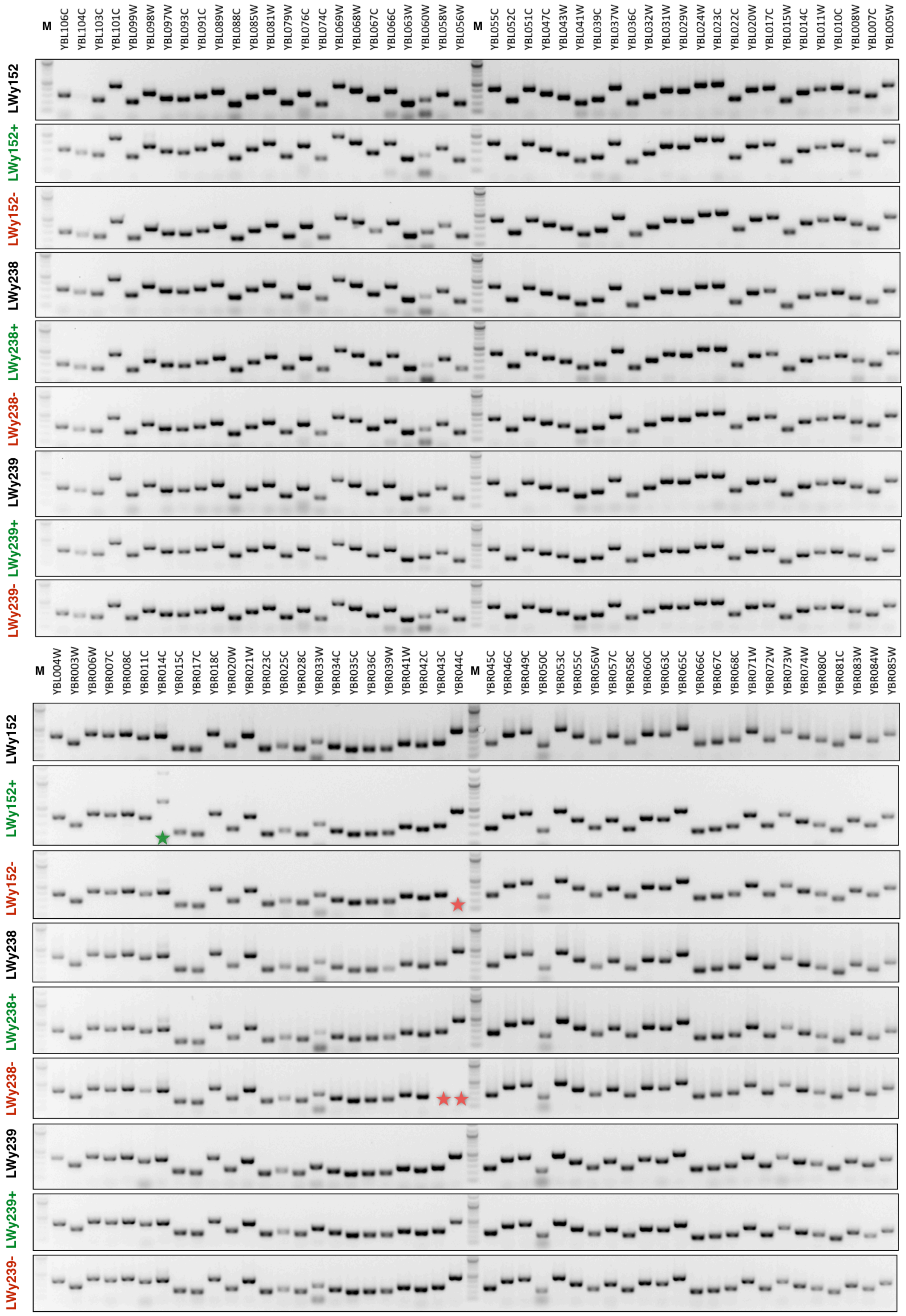
M
YBL106C
YBL104C
YBL103C
YBL101C
YBL099W
YBL098W
YBL097W
YBL093C
YBL091C
YBL089W
YBL088C
YBL085W
YBL081W
YBL079W
YBL076C
YBL074C
YBL069W
YBL068W
YBL067C
YBL066C
YBL063W
YBL060W
YBL058W
YBL056W
M
YBL055C
YBL052C
YBL051C
YBL047C
YBL043W
YBL041W
YBL039C
YBL037W
YBL036C
YBL032W
YBL031W
YBL029W
YBL024W
YBL023C
YBL022C
YBL020W
YBL017C
YBL015W
YBL014C
YBL011W
YBL010C
YBL008W
YBL007C
YBL005W
LWy152
LWy152+
LWy152-
LWy238
LWy238+
LWy238-
LWy239
LWy239+
LWy239-
M
YBL004W
YBR003W
YBR006W
YBR007C
YBR008C
YBR011C
YBR014C
YBR015C
YBR017C
YBR018C
YBR020W
YBR021W
YBR023C
YBR025C
YBR028C
YBR033W
YBR034C
YBR035C
YBR036C
YBR039W
YBR041W
YBR042C
YBR043C
YBR044C
M
YBR045C
YBR046C
YBR049C
YBR050C
YBR053C
YBR055C
YBR056W
YBR057C
YBR058C
YBR060C
YBR063C
YBR065C
YBR066C
YBR067C
YBR068C
YBR071W
YBR072W
YBR073W
YBR074W
YBR080C
YBR081C
YBR083W
YBR084W
YBR085W
LWy152
LWy152+
LWy152-
LWy238
LWy238+
LWy238-
LWy239
LWy239+
LWy239-

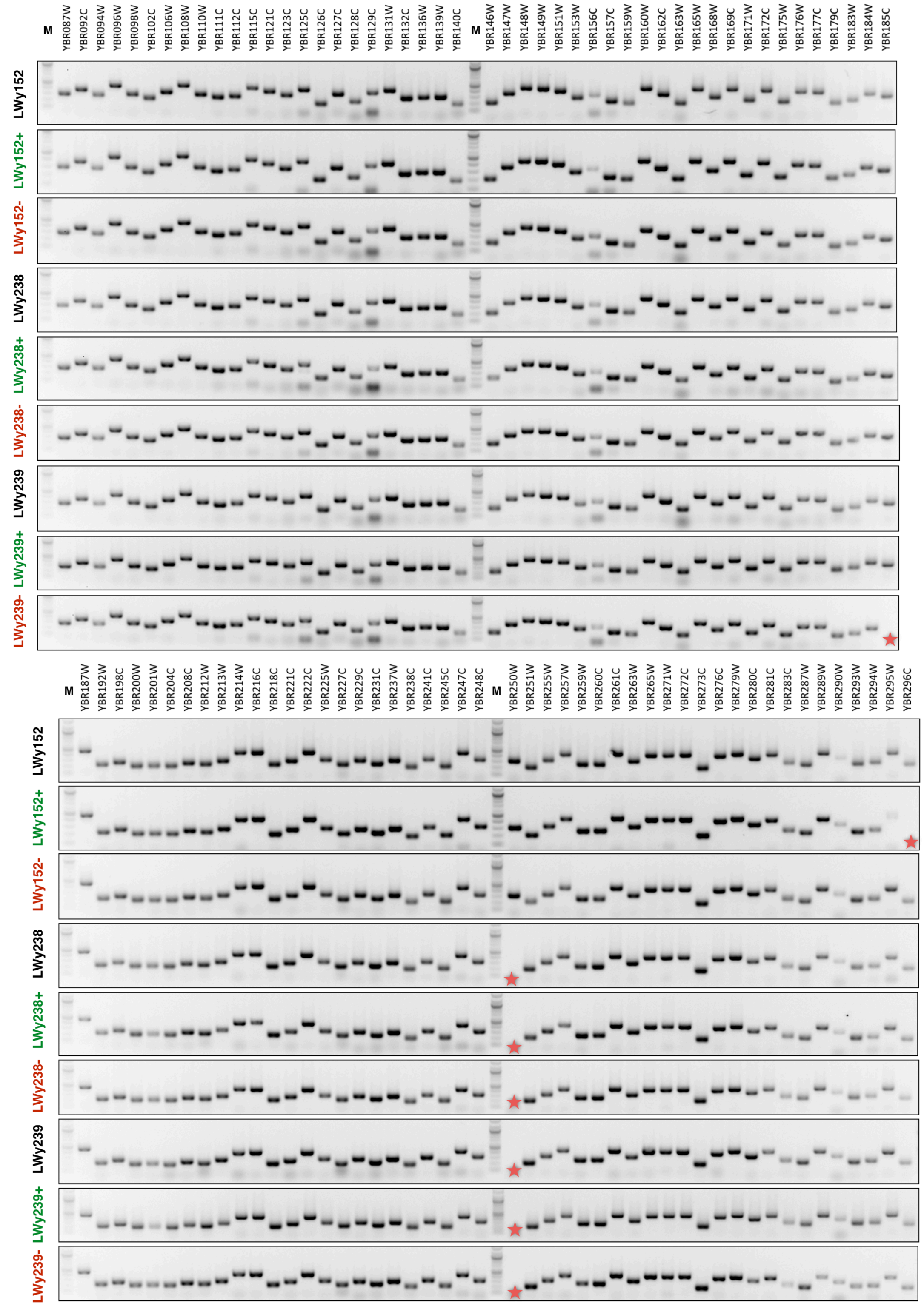

Supplementary Figure 9. **PCR tag analysis of continued SCRaMbLE variants with violacein pathway.** LWy152 has no missing tags; LWy152+ has a missing tag at *YBR296C* and a modified tag at *YBR014C*; LWy152- has a missing tag at *YBR044C*; LWy238, 238+, 238-, 239, 239+, 239- all have a missing tag at *YBR250W*; LWy238- has two extra missing tags at *YBR043C* and *YBR044C*; LWy239- has an extra missing tag at *YBR185C*. Red star indicates fragment deletion; green star indicates potential fragment duplication or other types of variation.

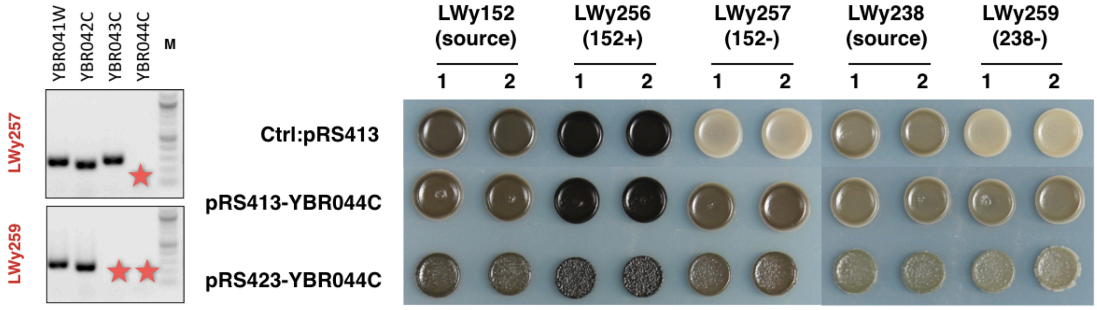

Supplementary Figure 10. *YBR044C* complementation experiment. LWy257 and LWy259 have the same *YBR044C* deletion. LWy256 and LWy259 complemented with pRS413-*YBR044C* recovered the phenotype as the LWy152 and LWy238 source strain respectively. Overproduction of *YBR044C* on pRS423 vector did not obviously improve the production of violacein but affected cell fitness instead.

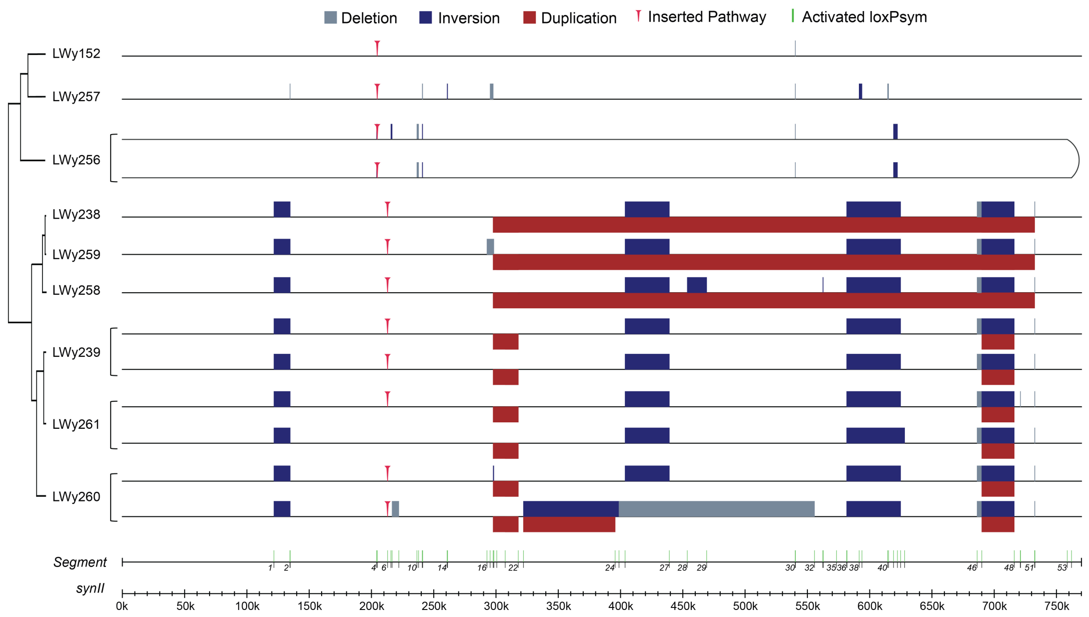

Supplementary Figure 11. SCRaMbLE events overview of strains with violacein. SCRaMbLE events are represented as rectangular bars with different color code. The gray bar represents deletion, the blue and brown indicate inversion and duplication. The red spike symbol indicates the SCRaMbLE-in locus of an exogenous violacein synthesis pathway. The activated *loxPsym* (green bars) is defined as the *loxPsym* site at the flanking of SCRaMbLE event. The cluster of strains illustrates the component difference on the position and length of SCRaMbLE event by hierarchical clustering method. The synII parental chromosome comprises 55 segments, numbered consecutively as shown, sectionalized by activated *loxPsym*. A curve joint in LWy256 represents a chromosome fusion event.

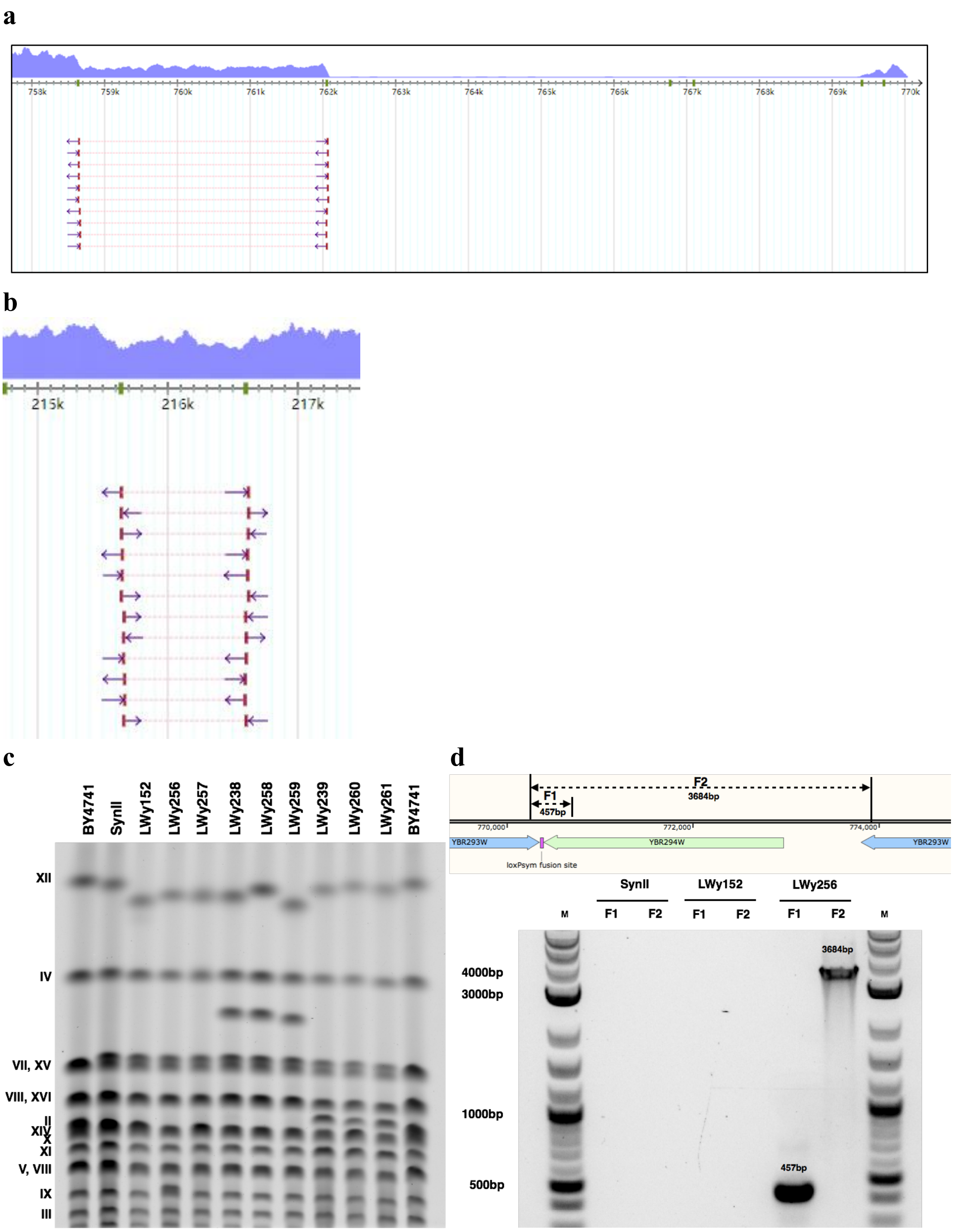

Supplementary Figure 12. Chromosome fusion and centromere inversion events in LWy256. (a) The split read map indicated that a near telomere sequence deletion start from 762 Kb since no reads could be found at this region. The reads mapped near 770 Kb were due to the presence of identical sequence at the other telomere of synII. The split reads between 758 Kb to 762 Kb indicates the chromosome fusion happened at the same end of synII, resulting an invert-connected fusion chromosome. **(b)** The split read map indicated that one of the two centromeres was inverted. **(c)** The PFGE result of chromosomes in all SCRaMbLEd strains. **(d)** PCR confirmation of chromosome fusion site in LWy256. The last gene in SynII *YBR294W* was reverted, followed with a whole reverted mirror sequence in LWy256.

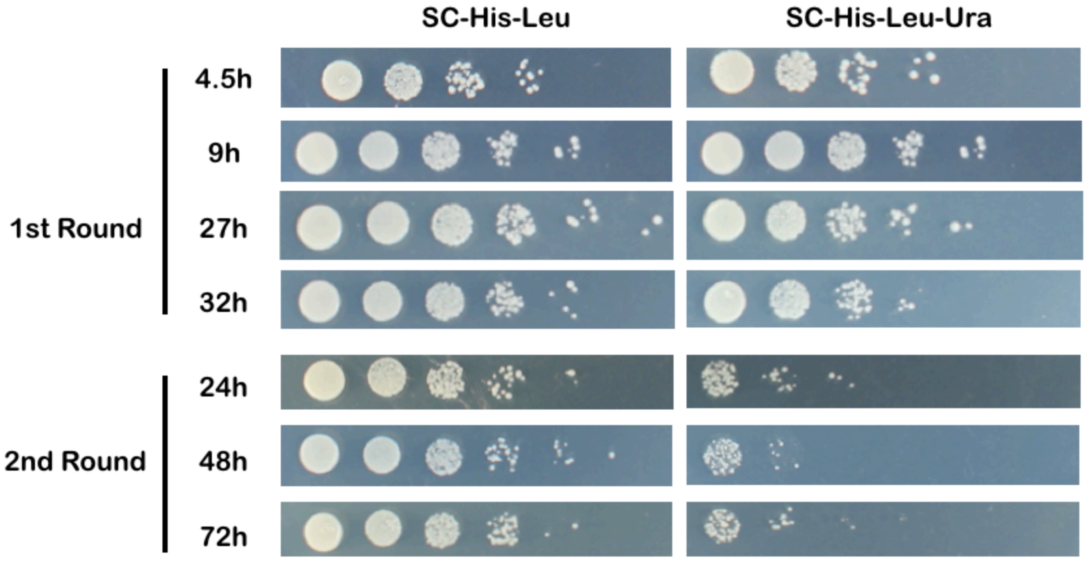

Supplementary Figure 13. Recombination induction test for DreEBD. In the first round of induction, samples with pSCW11-DreEBD were spotted at 4.5 h, 9 h, 27 h and 32 h. A second round of induction with fresh 1 μM β-estradiol was performed after 32h in the first round and continued for another 24 h, 48 h and 72 h. The induction effect started to show after 27 h induction; colony number continued to drop with longer induction as shown in 32 h induction spotting; colony number of second round 24 h induction have a more obvious drop, indicating a better induction with fresh estradiol after 32 h; colony number continued to drop till 48 h induction in the second round; no great colony number difference is observed between 48 h and 72 h induction in the second round.

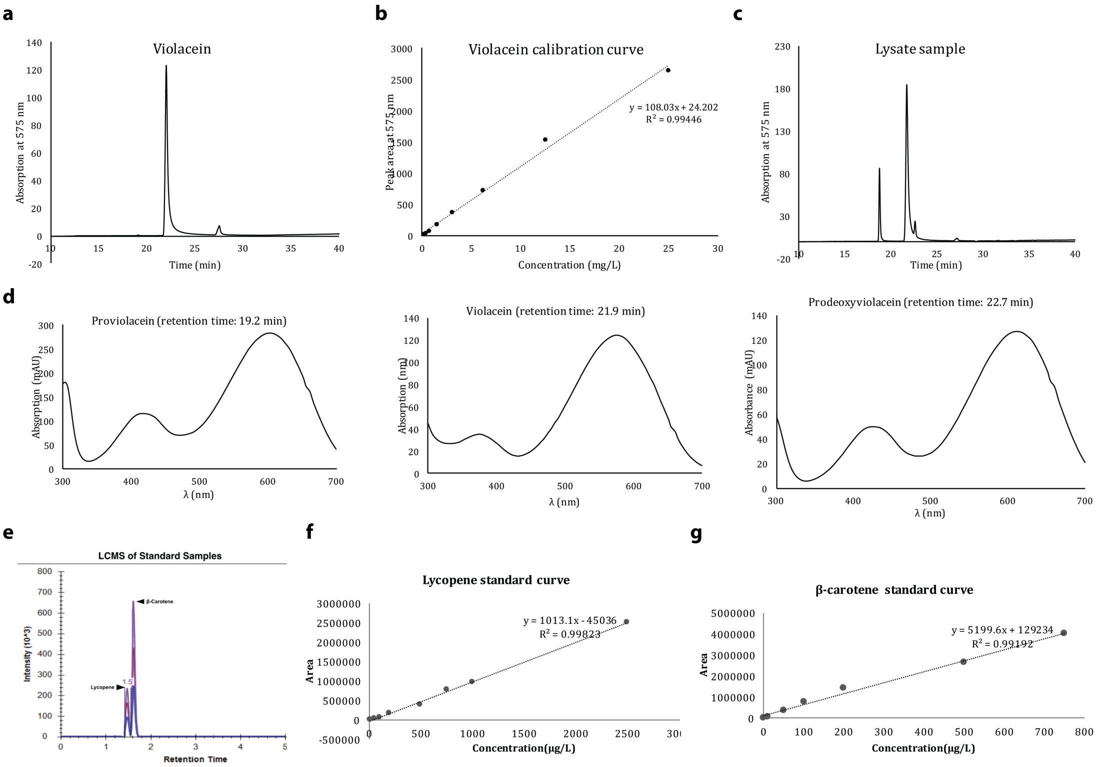

Supplementary Figure 14. Quantification of violacein and carotenoids. (a) HPLC chromatogram (575 nm trace) of commercial violacein (purity 87%). The retention time of violacein was 21.9 min. **(b)** HPLC calibration curve for violacein. **(c)** A typical HPLC chromatogram for an violacein extract. The largest peak (2nd) has the same retention time as violacein in the commercial sample and mass spectrometry showed the correct *m/z* for violacein $[M+H]^+ = 344$. Spectrum of reference violacein, max 575 nm. **(d)** Spectrum of peaks from the cultures. First peak, retention time 19. 2 min, max 602 nm; second peak, retention time 21.9 min, max 575 nm; third peak, retention time 22.7 min, max 612 nm. **(e)** LCMS chromatogram of commercial lycopene and β-carotene. **(f)** LCMS calibration curve for lycopene. **(g)** LCMS calibration curve for β-carotene.

Supplementary Tables

Supplementary Table 1. Promoters from YeastFab library

Number	System name	Promoter activity relative to pCYC1[5]
1	YGR192C_P	54.89
2	YHR174W_P	39.14
3	YFR053C_P	19.88
4	YFL014W_P	17.34
5	YGL030W_P	14.21
6	YGL038C_P	13.73
7	YFL026W_P	11.84
8	YHR010W_P	10.73
9	YHR021C_P	10.45
10	YFL039C_P	9.03
11	YHR141C_P	7.81
12	YGL037C_P	7.73
13	YFL034C-A_P	7.67
14	YGR189C_P	7.49
15	YHR179W_P	6.26
16	YHR214W_P	6.08
17	YHR097C_P	5.95
18	YHR008C_P	5.77
19	YGL032C_P	5.72
20	YFL031W_P	5.56
21	YGR280C_P	5.54
22	YGR279C_P	5.15
23	YGL005C_P	2.03
24	YGR270W_P	2.01
25	YGR295C_P	2.01

Supplementary Table 2. Composition of β-carotene and violacein pathway

Promoter	CDS	Terminator
5'UTR of *TDH3**	*CrtI*	3'UTR of *ACS2*
5'UTR of *PGK1*	*CrtE*	3'UTR of *ACS1*
5'UTR of *ACT1*	*CrtYB*	3'UTR of *ENO2*
5'UTR of *TDH3**	*VioA*	3'UTR of *ACS2*
5'UTR of *PGK1*	*VioB*	3'UTR of *ENO2*
5'UTR of *ACT1*	*VioC*	3'UTR of *ACS1*
5'UTR of *RPS2*	*VioD*	3'UTR of *CIT1*
5'UTR of *ZEO1*	*VioE*	3'UTR of *FUM1*

* The promoter for *CrtI* or *VioA* are present in pathway SCRaMbLE-in device. In the in vitro promoter integration experiment, the upstream of *CrtI* or *VioA* were corresponding recombination sites instead.

Supplementary methods

Strains and media

Bacterial strain BL21 (DE3) was used for recombinase expression; ccdB Survival™ 2 (ThermoFisher scientific) was used for *ccdB* containing plasmid cloning. Yeast strain BY4741 was used for β-carotene and violacein expression after *in vitro* promoter integration. Yeast strain YSy115 with synthetic chromosome II (*SynII* v1.3) was used for pathway SCRaMbLE-in. LB with Ampicillin (100 μg/mL), LB with Kanamycin (50 μg/mL) were used for bacterial culture and DNA cloning. SC-Leu plates supplemented with 5-FOA (1mg/mL) were used for pathway SCRaMbLE-in counter selection.

Gene circuit construction

Recombinase expressing circuit for purification. Cre was amplified by PCR from a CreEBD expression vector[1]; Dre was identified from phage D6, the coding sequence was obtained from DDBJ/EMBL/GenBank database with the accession number of AY753669, and Dre was purchased as gBlock from Integrated DNA Technologies [2]; VCre was identified from *Vibrio sp. 0908*, the sequence was obtained from GenBank with the accession number of ABX77110.1, and VCre was purchased from Twist Biosciences. All recombinase sequences were flanked by NcoI and XhoI restriction sites with start and stop codons removed. The cassettes were cloned into the pET-28a vector (Novagen) to be expressed in frame with a C-terminal His_6 tag.

Recombination test circuits. Basic function test of recombinases: pWL085, pWL008 and pWL022. Ura3 cassette was tailed with *loxP*, *rox* or *Vlox* sites by PCR using KAPA HiFi PCR Kit (KAPABIOSYSTEMS) and cloned into pRS413 vector by Gibson assembly. Excision rate: *RFP* gene was tailed with *loxP*, *loxP* mutant sites and was cloned into pRS415 vector by Gibson assembly. Integration rate: for excision part, insert *ccdB* cassette to excision rate test device by Gibson assembly; for integration part, *RFP* cassette was tailed with *loxP*, *loxP* mutant sites, *rox* or *Vlox* sites by PCR using KAPA HiFi Kit and cloned into HCKan_P vector by Gibson assembly. The constructs went through a round of in vitro recombination, leaving only one recombination site on the vector.

Promoter loading vector. BsmBI sites were removed from the pUC19 plasmid by PCR mutagenesis[3]. *URA3*-BsmBI-*RFP*-BsmBI cassettes flanking by *loxJTZ17* and *Vlox* were assembled with SmaI linearized pUC19 by Gibson assembly.

Pathway HO integration vector. The first 500 bp of *HO* gene were tailed with two BsaI sites at each end and a SmaI site at 3’ end; *HIS3* gene and *HO* upstream 500bp were flanked with two BsaI sites. The three fragments were amplified by Phusion PCR and purified by PureLink® PCR Purification Kit. HCKan_P, *HO* first 500 bp, *HIS3*, *HO* upstream 500 bp were assembled by BsaI Golden Gate assembly. A *RFP* gene flanked by two YeastFab compatible BsaI sites was inserted into SmaI site by Gibson assembly.

Promoter integration pathway circuit. Construction of promoter acceptor for a pathway includes three rounds of YeastFab standard Golden Gate assembly (SM Figure 2). Promoters and *RFP* cassette flanked by *loxJT15* or *Vlox* sites were cloned into HCKan_P vector by BsaI Golden Gate assembly. Pathway synthesis genes were cloned into HCKan_O vector. Terminators were cloned into HCKan_T vector. Promoter or

recombination site flanking *RFP*, pathway synthesis gene and terminator were assembled to a POT acceptor vector by BsmBI Golden Gate assembly. The recombination site *RFP* containing unit went through one step in vitro recombination to get rid of *RFP*. Then the POT units were assembled into the *HO* integration acceptor vector by BsaI Golden Gate assembly as a final step.

Pathway SCRaMbLE-in circuit. RFP cassette flanked by *loxPsym* sites was inserted to pRS416 vector by Gibson assembly. *LEU2* marker was put into a proper POT vector to assembly with genes of pathway by YeastFab Golden Gate assembly.

Golden Gate Assembly

BsaI based Golden Gate Assembly. In a 20μL reaction, mix 1μL BsaI, 1μL T4 ligase, 2μL 10x T4 ligase buffer, 0.2μL 100X BSA, equal molar DNA fragments with a total quantity of 1μg and make up to 20μL with ddH_2O. Incubate in a thermocycler with the following program: 10-30 cycles of (37°C for 5 minutes, 16°C for 10 minutes), then 50°C for 10 minutes, 80°C for 5 minutes.

BsmBI based Golden Gate Assembly. In a 10μL reaction, mix 1μL 10 ×T4 ligase Buffer (NEB), 0.1μL 100X BSA, 0.5μL BsmBI, equal molar of DNA fragments with a total quantity of 500ng and adjust the volume to 9μL with dH_2O. Incubate in a thermocycler with the following program: 55°C for 90min, add 0.5μL T4 DNA ligase, 10-30 cycles of (37°C for 5 minutes, 16°C for 10 minutes), then 55°C for 10 minutes, 80°C for 5 minutes.

Gibson Assembly

15 μL Gibson master mix (4μL 5x ISO buffer, 0.16μL T5 Exo (1/20), 0.25μL Phusion Pol, 2μL 160μL of 40(U/μL) Taq Ligase, 8.59μL H_2O) were mixed with equal molar of purified DNA fragments to final volume of 20μL. Keep in 50 °C for 1h.

Violacein calibration curve

Violacein was purchased from Sigma-Aldrich (V9389-1MG). Solutions with various concentration (25, 12.5, 6.25, 3.13, 1.56, 0.78, 0.39 and 0.20 mg/L) of commercial violacein were prepared in methanol and analyzed by HPLC. Retention time of violacein was determined to be 21.9 min and the area under the peak was determined to generate the calibration curve (Supplementary Fig. 14a, 14b).

Determination of the amount of violacein from extracts

The samples obtained from the lysates were analyzed by HPLC with the same method and equipment as above. Under these conditions, the 575 nm trace showed three peaks with different retention times (19.2, 21.9 and 22.7 min) (Supplementary Fig. 14c). These peaks could be assigned to different compounds on the pathway of violacein production by its absorption spectrum[4]. The first peak corresponds to proviolacein with a retention time of 19.2 min, and a maximum in absorption at 602 nm. The second peak corresponds to violacein with a retention time of 21.9 min, and a maximum in absorption at 575 nm. The third peak corresponds to prodeoxyviolacein with a retention time of 22.7 min, and a maximum in absorption at 612 nm (Supplementary Fig. 14d).

Lycopene and β-carotene calibration curve

Lycopene and β-carotene was purchased from Sigma-Aldrich. Solutions with various concentration (2.50, 1.00, 0.75, 0.50, 0.20, 0.10 and 0.05 mg/L of commercial lycopene and 0.75, 0.50, 0.20, 0.10, 0.05 and 0.01 mg/L of commercial β-carotene) were prepared in isopropanol and analyzed by LCMS. Retention time of lycopene was determined to be 1.5 min and the area under the peak was calculated to generate the calibration curve; β-carotene was determined to be 1.7 min and the area under the peak was calculated to generate the calibration curve (Supplementary Fig 14e, 14f, 14g).

Supplementary References

1. Lindstrom DL, Gottschling DE. The mother enrichment program: a genetic system for facile replicative life span analysis in Saccharomyces cerevisiae. *Genetics* **183**, 413-422, 411SI-413SI (2009).

2. Sauer B, McDermott J. DNA recombination with a heterospecific Cre homolog identified from comparison of the pac-c1 regions of P1-related phages. *Nucleic Acids Res* **32**, 6086-6095 (2004).

3. Mitchell LA*, et al.* Multichange isothermal mutagenesis: a new strategy for multiple site-directed mutations in plasmid DNA. *ACS Synth Biol* **2**, 473-477 (2013).

4. Lee ME, Aswani A, Han AS, Tomlin CJ, Dueber JE. Expression-level optimization of a multi-enzyme pathway in the absence of a high-throughput assay. *Nucleic Acids Res* **41**, 10668-10678 (2013).

5. Guo Y*, et al.* YeastFab: the design and construction of standard biological parts for metabolic engineering in Saccharomyces cerevisiae. *Nucleic Acids Res* **43**, e88 (2015).
