# Supplementary material for: Rapid pathway prototyping and engineering using in vitro and in vivo synthetic genome SCRaMbLE-in methods
Source: Nat Commun. 2018 May 22;9:1936. doi: 10.1038/s41467-018-04254-0 (PMC5964202; doi:10.1038/s41467-018-04254-0)
Supplement: Supplementary file 2 — Description of Additional Supplementary Files [file 41467_2018_4254_MOESM2_ESM.pdf]

# Description of Additional Supplementary Files

File Name: Supplementary Data 1
Description: Detailed information of genotype variations of the SCRaMbLEd strains and annotations of the affected coding sequences, plus recombinase sequences, plasmid and strain tables.
